# Molecular Dynamics of CYP2D6 Polymorphisms in the Absence and Presence of a Mechanism-Based Inactivator Reveals Changes in Local Flexibility and Dominant Substrate Access Channels

**DOI:** 10.1371/journal.pone.0108607

**Published:** 2014-10-06

**Authors:** Parker W. de Waal, Kyle F. Sunden, Laura Lowe Furge

**Affiliations:** Department of Chemistry, Kalamazoo College, Kalamazoo, Michigan, United States of America; University of Copenhagen, Denmark

## Abstract

Cytochrome P450 enzymes (CYPs) represent an important enzyme superfamily involved in metabolism of many endogenous and exogenous small molecules. CYP2D6 is responsible for ∼15% of CYP-mediated drug metabolism and exhibits large phenotypic diversity within CYPs with over 100 different allelic variants. Many of these variants lead to functional changes in enzyme activity and substrate selectivity. Herein, a molecular dynamics comparative analysis of four different variants of CYP2D6 was performed. The comparative analysis included simulations with and without SCH 66712, a ligand that is also a mechanism-based inactivator, in order to investigate the possible structural basis of CYP2D6 inactivation. Analysis of protein stability highlighted significantly altered flexibility in both proximal and distal residues from the variant residues. In the absence of SCH 66712, *34, *17-2, and *17-3 displayed more flexibility than *1, and *53 displayed more rigidity. SCH 66712 binding reversed flexibility in *17-2 and *17-3, through *53 remained largely rigid. Throughout simulations with docked SCH 66712, ligand orientation within the heme-binding pocket was consistent with previously identified sites of metabolism and measured binding energies. Subsequent tunnel analysis of substrate access, egress, and solvent channels displayed varied bottle-neck radii. Taken together, our results indicate that SCH 66712 should inactivate these allelic variants, although varied flexibility and substrate binding-pocket accessibility may alter its interaction abilities.

## Introduction

Cytochrome P450s represent a superfamily of heme-thiolate-containing enzymes responsible for metabolism of endogenous substrates such as steroids, fatty acids, and eicosanoids as well as xenobiotics including a large majority of pharmaceutical agents [1]. CYP2D6 is a xenobiotic metabolizing CYP responsible for metabolism of ∼15% of pharmaceutical drugs including many with narrow therapeutic indices such as those for regulation of blood pressure and anti-psychotics [2], [3]. The only CYP that metabolizes more pharmaceuticals is CYP3A4.

Unlike CYP3A4, however, CYP2D6 is expressed as over 100 different allelic variants resulting in a large range of enzymatic activity over different individuals [4], [5], [6]. The Human Cytochrome P450 Allele Nomenclature Committee (http://www.cypalleles.ki.se/) has an up-to-date listing of CYP polymorphisms and a recent review of known clinical phenotypes of some alleles has been published [7], [8]. CYP polymorphisms result in variations in plasma drug concentrations and half-life of drugs *in vivo*. Allelic variants may result in null phenotype, reduced activity, altered substrate selectivity, altered sites of metabolism, and even increased catalytic efficiency with some drug substrates [7], [8]. For instance, individuals with multiple copies of CYP2D6 rapidly clear drugs metabolized by CYP2D6 and are referred to as ultra-metabolizers (UMs) while those who have reduced or null CYP2D6 activity are referred to as poor metabolizers (PMs) [7]. All others are considered intermediate (IM) or extensive metabolizers (EMs) reflecting the large variation in CYP2D6 activity among individuals [7]. Allelic variants of CYP2D6 present a major area of concern for pharmaceutical drug metabolism and adverse drug interactions due to wide variability in drug metabolism activity caused by the presence of variants [4].

In addition to the challenge of categorizing CYP2D6 polymorphisms and drug efficacy, CYP2D6 metabolism can also be severely affected by mechanism-based inactivation (MBI). Mechanism-based inactivators are ligands for CYPs that are metabolized to reactive electrophiles. The electrophiles can then become covalently bound to the enzyme or the heme moiety, or cause enzyme-heme covalent adduction [9]. Frequency of formation of reactive electrophiles depends on the partion ratio for the inactivator, e.g. number of turn over events minus one. Adverse drug reactions resulting from MBI result in patient phenotypes similar to poor-metabolizers and severe side effects can result [9]. Little is known about the interaction between CYP polymorphisms and inactivators. Understanding of how polymorphisms cause changes in enzyme activity and application to inactivators will lead to better prediction of clinical outcomes in the era of personalized medicine as well as allow better predictions of structure-activity relationships between CYPs and various substrates. Furthermore, understanding interactions of MBIs with polymorphic forms of CYPs will lend insight into the basic chemical structures and physical interactions that lead to MBI events.

Crystal structures of many of the xenobiotic metabolizing human CYPs have been solved [10]. These structures typically present reference forms, termed *1, for CYPs, e.g. the most common CYP variant found in the general population. In the star allele nomenclature, *1 represents the consensus sequence while other novel variants are ordered by a unique number (for more information on the star allele nomenclature, see [11]). For crystallographic studies there are frequently mutations introduced to facilitate solubility and crystallization (e.g. truncation of the N-terminal domain). In order to understand the effects of allelic variants on CYP structure, substrate binding, access/egress channels, and other physical characteristics, molecular dynamics models of allelic variants can be compared to *1 structures [12], [13], [14], [15].

**Figure 1:**
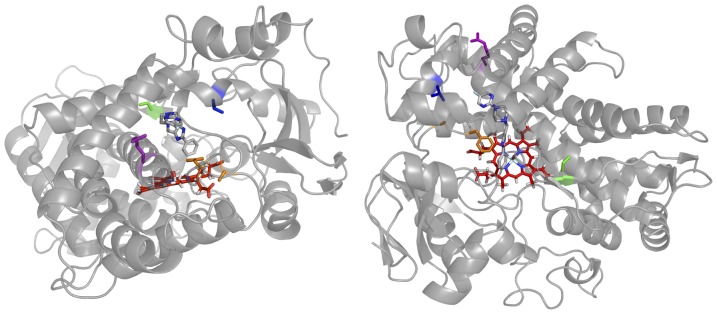
Location in CYP2D6*1 of the amino acid variants (the structure is shown in two views). *34 has a single mutation at R296C (purple) on helix I and distal to the active site. *17-2 has the R296 mutation as well as T107I (blue) while *17-3 also has the S486T mutation (green), but distal to the active site. *53 has two mutations in SRS1 at F120I and A122S (orange) that are near the active site. Heme is shown in red.

Four polymorphic forms of CYP2D6 (*34, *17-2, *17-3, and *53) were chosen for this study as a preliminary examination of the use of molecular dynamics models in understanding polymorphisms and inactivation (Table 1 and Figure 1). Variants were chosen based on clinical relevance (*17-2 and *17-3), mutational similarity (*34, *17-2, *17-3), and functional activity (*53) (*vide infra*).

**Table 1:** Allelic Variants of CYP2D6 Analyzed in this Study^∧^.

Allele	Alterations	Location (s)	Functional Significance	References
*34	R296C	αI	metabolic products vary from expected	[58]
*17-2	R296C, T107I	αI, αB′	activity reduced to ∼20%	[16]
*17-3	R296C, T107I, S486T	αI, αB′, β4-2	activity reduced to ∼20%	[7]
*53	F120I, A122S	B-C loop	possible UM	[6]

^∧^ amino acid alterations and functional significance as compared to allelic form CYP2D6*1.

The variant *34 has one altered amino acid - an R296C mutation near the start of helix I near substrate recognition site 4 (SRS4) that is located ∼20 Å from the heme iron. The R296C amino acid change is a common variation in combination with other mutations in allelic variants of CYP2D6. *17-2 has the R296C mutation as well as a T107I mutation in helix B′ near SRS1, also ∼20 Å from the heme iron. *17-3 has a third additional mutation, S486T, in sheet β4-2 near SRS6 at ∼14 Å from the heme iron. The difference between *17-2 and *17-3 is two or three mutations, respectively, hence the designations -2 and -3; no functional activity differences have been noted for the two variants [16]. The series of *34, *17-2, and *17-3 variants allows for analysis of the effects of increasing numbers of mutations to be analyzed as well as the effects of distal mutations on the active site.

One additional variant, *53, was also used in our analysis due to its characterization as a possible UM [6]. *53 has two amino acid changes: F120I and A122S. Both of these mutations are located in the B-C loop region and SRS1. These amino acids are near the active site and position 120 is only ∼4.5 Å from the heme iron. The B-C loop region is known to have a role in substrate recognition and binding [17]. F120 is part of the active site architecture (the “arch” portion of a right foot shape of the active site of CYP2D6 as described by Rowland et al. [18]) and is believed to interact with aromatic substrates.

Previous studies have shown that allelic variants *34, *17-2, and *17-3 (mutations distal from the active site) display ∼80% reduced activity compared to *1 [7]. *34 has further been shown to form different major products than *1 during metabolism of some drugs (e.g. anandamide [19]). For *17-2 and *17-3 variants, the mutations that lead to lower activity have measurable effects on *K*_m_ (increased) for some substrates (e.g. codeine [16], [20]). Conversely, *53 (two mutations near active site) is considered a possible UM with lower *K*_m_ for some substrates and resulting higher enzyme efficiency (e.g. bufuralol and dextromethorphan [6]).

**Figure 2:**
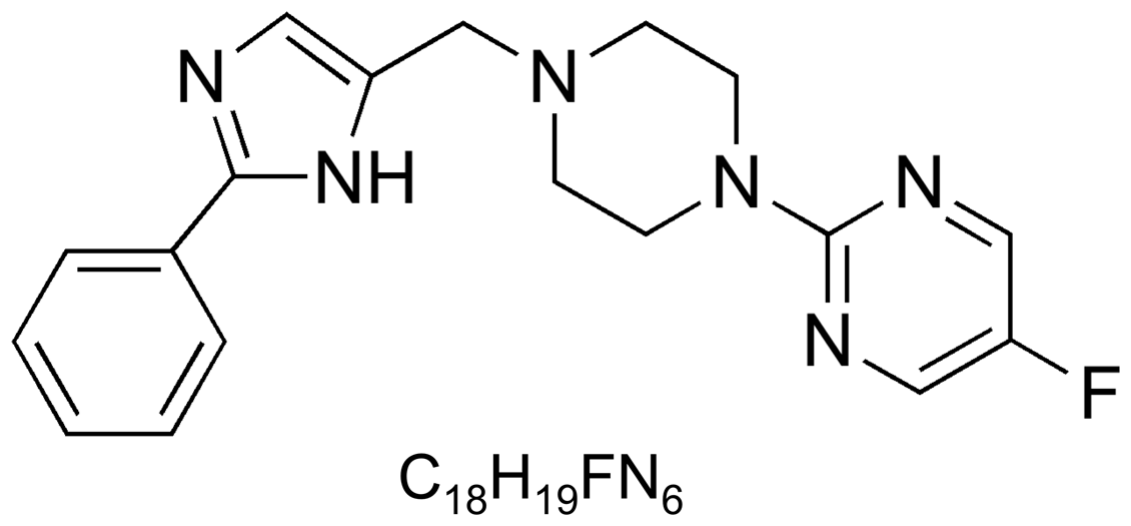
The structure of SCH 66712 consists of phenyl, imidazole, piperazine, and fluorinated heteroaromatic rings. SCH 66712 is metabolized by CYP2D6 to four mono-oxygenation products. One product is formed by oxygenation of the phenyl ring (most likely on the *para*-carbon) and three products are formed by oxygenation at sites on the piperazine and/or heteroaromatic rings, as previously described [21].

Our group has previously established SCH 66712 as a potent mechanism-based inactivator (MBI) of CYP2D6*1 (Figure 2) [21], [22]. In addition, we determined the sites of metabolism on SCH 66712 by CYP2D6 and predicted the route for inactivation [21]. While some kinetic data for interaction of some ligands with CYP2D6 allelic variants exists, how SCH 66712 or other ligands and MBIs interact with CYP2D6 allelic variants is not known. Since SCH 66712 is both a ligand and inactivator, it is well suited for use in analysis of CYP2D6 allelic variant interactions with ligands. Furthermore, tunnel analysis with allelic variants of CYP2D6 has not previously been performed, but the strategy may allow greater understanding of some observed activities and allow prediction of susceptibility to inactivation. Thus, the aim of this study was to use computational approaches to explain possible interactions between polymorphisms of CYP2D6 and SCH 66712 that can lead to differences in susceptibility to inactivation.

By use of molecular dynamics techniques, the present study showed differences in conformational dynamics of a subset of CYP2D6 variants that could explain observed phenotypes and possible interaction with the inactivator SCH 66712. Mutations either distal or proximal to the active site generated changes in protein flexibility without affecting global protein folding. In addition, the presence of the ligand, SCH 66712, in the active site caused changes in protein flexibility that might affect substrate egress channel pathways and further explain the differences in enzyme activities among the allelic variants. Overall, our findings predict similar susceptibility to inactivation by SCH 66712 for each variant.

## Methods

### Model preparation

The initial model structure for molecular dynamics (MD) simulations was constructed from the 2.8 Å resolution X-ray structure of CYP2D6 bound to prinomastat (PDB ID: 3QM4) [23]. Only chain A and its corresponding heme cofactor were used as the base template for this study; crystallographic waters and prinomastat were removed. The 3QM4 structure was used since it was crystallized with primomastat bound and, in conjunction with our compound I heme parameters (*vide infra*), better simulates the hexa-coordinated iron of the heme that is only found during catalysis. There is also a 2.1 Å resolution structure of CYP2D6 (3TBG) that contains two thioridazines bound above the active site (each at 370 da and with bulky fused six-member rings). The presence of two thioridazine ligands in 3TBG opens the active site cavity more than in 3QM4 and merges substrate access channels [9]. Since binding of only a single ligand of approximately the same shape and size of prinomastat and analysis of substrate access/egress channels were key elements in the current study, the 3QM4 structure was used instead of 3TBG. Finally, the substrate-free structure (PDB ID: 2F9Q) lacked sufficient electron density for assignment of residues 42-51 in the N-terminal region and 229–239 of the F-G loop; the 2F9Q further contained a polymorphic V374M substitution and two additional mutations, L230D and L231R, introduced to increase solubility [18]. While the N-terminal region of CYP2D6 was modified by truncation to increase expression and solubility, and a histidine tag introduced for purification purposes on the C-terminal end, CYP2D6 in the 3QM4 structure otherwise represents CYP2D6*1.

Backbone dependent rotamers were added to *1 to create *34 (R296C), *17-2 (T107I, R296C), *17-3 (T107I, R296C, S486T), and *53 (F120I, A122S) using UCSF Chimera package [24]. Protonation states of histidines, glutamic, and aspartic acids for all polymorphisms were determined at pH 7.0 by the PDB2PQR [25], [26] webserver using PROPKA [27]. To create ligand bound complexes, SCH 66712 was docked into the binding pocket using AutoDock Vina [28]. While a crystal structure of CYP2D6 bound to SCH 66712 has yet to be elucidated, docking positions for simulation are consistent with previously observed metabolism mechanisms and presumed orientation for mechanism-based inactivation, namely phenyl ring oriented toward the heme iron [21]. Priority was also given for pi-pi stacking geometry of one of the aromatic rings of SCH 66712 with F120 and interaction of one of the basic nitrogens of SCH 66712 with D301 or E216 [22].

### Simulation protocol

Setup and simulation was performed as follows for all systems using pmemd.cuda (hybrid single/double precision) [29] in the AMBER 12 (Bugfix 2) suite with the AMBER99SB and GAFF force fields [30], [31]. To model the catalytically active oxy-ferryl-species [(Fe^IV^ = O)^+•^], quantum mechanically derived parameters for resting high spin Compound I were applied as described and provided to us by Shahrokh et al. [32]. RESP charges for SCH 66712 were derived by the RESP-A1A charge model in Gaussian 2009 using the R.E.D. Server [33]. The system was solvated in a 10 Å pad of TIP3P waters and neutralized. Additional NaCl ions were randomly added for a final concentration of 150 mM to mimic physiological conditions.

To reduce steric clashes between the solvent and protein, before simulation a 10,000 step combined steepest and conjugate gradient energy minimization was performed with harmonic restraints of 25 kcal/mol-Å^2^ placed on all protein backbone atoms. A subsequent full system 10,000 step energy minimization was performed without harmonic restraints. Following energy minimization the system was linearly heated in the canonical NVT ensemble (constant number of particles, N; volume, V; temperature, T) to 300.0 K using the Langevin thermostat with a collision frequency of 5.0 ps^-1^ and harmonic restraints of 4 kcal/mol-Å^2^ on all backbone atoms over 250 ps. To equilibrate pressure and volume, three 250 ps simulations were performed in the isothermal-isobaric NPT ensemble (constant number of particles, N; pressure, P; temperature, T) lowering harmonic restraints on all backbone atoms by 1 kcal/mol-Å^2^ each time with isotropic position scaling using the weak-coupling Berendsen barostat, a coupling constant of 1 ps^-1^, and a target pressure of 1 atm. A final 250 ps NPT simulation was performed without harmonic restraints and a Langevin collision frequency of 2 ps^−1^. Similar to heating, 100 ns production runs were performed in the NVT ensemble but with a Langevin collision frequency of 1 ps^−1^ and without harmonic restraints. To avoid synchronization effects, a randomize seed, calculated from the wall clock, provided starting velocities for all simulations. The time-step for all simulations was 2 fs and all hydrogen atoms were constrained with the SHAKE algorithm with a tolerance of 1*10^−5^. Long-range electrostatic interactions were calculated using the Particle Mesh Ewald algorithm [34] with a cutoff of 9 Å. All other parameters were set to default values in AMBER in order to obtain a model of the best fit. Simulations were performed on the Stampede HPC system in the Texas Advanced Computing Center at the University of Text using a start-up allocation from the Extreme Science and Engineering Discovery Experience (XSEDE) interface.

We note that in the initial simulation for *1, SCH 66712 quickly flipped orientation, placing the heteroaromatic toward heme (data not shown). SCH 66712 stayed in the flipped orientation and moved into a pocket ∼12 Å above the ferryl oxygen, a binding distance not adequate for metabolism, for the duration of the simulation. Ligand behavior of this type was not seen with the other allelic variant simulations. We wondered if the results with *1 were due to the ligand assuming a binding free energy minimum that could not be overcome as the simulation continued over time. To confirm, we ran four additional simulations with SCH 66712 bound to *1 with each simulation starting with the same initial docking pose for SCH 66712 for a total of five simulations of *1 interaction with SCH 66712. Of the five simulations, two (1 and 3) resulted in the ligand locking quickly into a binding orientation away from the active site while the other three (2, 4, and 5) had the ligand staying within ∼3 Å of the active site and in a conformation compatible with metabolism. Simulations 2, 4, and 5 generated orientations and binding distances consistent with metabolism. The root mean square deviation (RMSD) equilibrations further showed that simulations 2, 4, and 5 were in greater equilibrium than simulations 1 and 3, the unproductive binding modes (data not shown). Analysis of root mean square fluctuation (RMSF) for simulations 2, 4, and 5 were similar (Figure S1) and Simulation 5 was used in all subsequent analyses.

### RMSD and RMSF

RMSD was calculated over the 100 ns production simulation for all backbone atoms (C_α_, C, N) at 10 ps intervals using cpptraj. A rolling average over 100 ps intervals was then applied to reduce noise. The RMSF of all backbone atoms was subsequently calculated over the latter half, 50–100 ns, of the production simulation. Corresponding RMSF structures were prepared by populating the B-Factor column in the pdb of the CYP2D6*1 with SCH 66712 simulation RMSF values using PBDEditor; the RMSF values were then visualized using PyMOL.

### Ligand Binding Clustering

Stable states of the ligand binding were identified via principal component analysis (PCA) where PC1 and PC2 measured the distance from the fluorine and phenyl ring end of SCH 66712, respectively, to the heme-coordinated oxygen (Compound I). Binding stability and populations were assessed via RMSD calculations for each ligand as described above.

### Calculation of Binding Free Energy

Relative ligand binding energies were calculated by Molecular Mechanics/Poisson Boltzmann Surface Area using MMPBSA.py [35], in 1 ns intervals over the last 20 ns of production simulation (80–100 ns) sampling over the previously identified SCH 66712 stable binding populations. To accurately describe the binding cavity environment containing the heme-coordinated oxygen and two positively charged Asp and Glu residues, an internal dielectric constant of 3 was applied as tested by Hou et al. [36]. Additionally nonpolar solvation energy was calculated as described by Tan et al. [37]. The MM/PBSA technique has been extensively described elsewhere [13], [38], [39], [40], [41].

### Tunnel analysis

Snapshots of each variant with no ligand bound were taken at 100 ps intervals over the 100 ns simulation run time generating a total of 1000 snapshots used in tunnel analysis. CAVER 3.0 software was used for analysis of substrate access and egress channels [42]. Using C programming language, the coordinates of the oxygen and iron of Compound I were identified and the starting point for CAVER analysis was set 4 Å above the oxygen in Compound I (Scheme S1). The probe radius was set to 0.9 Å and clustering threshold was initially set at default value of 4.0 but in final analysis ranged from 2.5 to 4.0. The bottleneck heat map range was 0.9–1.5 Å and the profile heat map range was 0.9–2.0 Å. Seed was set to 1 to ensure consistent results. All other parameters were set to default values as listed in the CAVER user guide version 3.0 and included: shell radius (3), weighting coefficient (1) for tunnel clustering, bottleneck contact distance (3), the number of approximating balls (12), max distance for the calculation starting point from the initial starting point (3), and desired radius (5) for the closest Voronoi vertex to the initial starting point. Resulting tunnels were identified and visualized in PyMOL. Heat maps were generated in R.

### Molecular Visualization

All molecular structures were produced using PyMOL Molecular Graphics System, Version 1.3 (Schrödinger, Portland, OR).

### Graphing

All graphs were prepared in the R Project for Statistical Computing (http://www.r-project.org/).

## Results

### System Convergence

Equilibration of the enzyme structures in MD simulations was checked by the root mean square deviation (RMSD) measurements for each simulation system (Figure S2). Deviations were measured over 100 ns. Forms *1 and *53 plateaued by ∼15 ns, with *34, *17-2 and *17-3 converging at ∼50 ns. The last 20 ns of the simulation trajectories were used in subsequent analyses unless otherwise stated.

### Comparison of *1 Model with 3QM4 Crystal Structure

Our model was built from the prinomastat bound crystal structure of CYP2D6 (3QM4) as described in the Methods. To confirm validity of our simulated model, the *1 model was superimposed on the 3QM4 structure with ligands bound (Figure S3). The overlay shows agreement between the crystal structure and the *1 model with RMSD of 1.724 Å. The overall structural fold was the same and secondary structural elements aligned. Differences noted include the appearance of helix A′ in the *1 model, though it did not form in the crystal structure 3QM4 (nor in the no ligand bound crystal structure, 2F9Q). Also, helix F′ in the *1 model was displaced ∼3.5 Å as was the connected F-G loop region; however, the distance between helix F′ and the region of helix A′ of the *1 model was proportional to the width in 3QM4 (∼10 Å). Finally, helix G″ was shorter in the *1 model (only one turn) and displaced ∼10 Å from its location in the 3QM4 crystal structure. These differences reflect the regions of CYPs known to be more flexible [43], [44] and the greater range of motion allowed in the molecular dynamics simulations versus crystal packing interactions observed by others [44].

With regard to ligand positioning, superposition of the *1 model with SCH 66712 docked in the active site onto the 3QM4 structure with bound prinomastat showed small overall displacement (<1 Å) of the central part of the ligands. However, at the heme iron, the SCH 66712 phenyl ring is displaced ∼4.7 Å relative to the position of the heteroaromatic ring of prinomastat (Figure S3B). The phenyl group of SCH 66712 produces a type I binding spectrum [21] and does not contain a nitrogen while the ring in prinomastat has a nitrogen and non-bonding electrons that coordinate tightly to the heme iron in the 3QM4 structure and produce a type II binding spectrum [23].

### Molecular Dynamics Simulations of Ligand-Free CYP2D6 Variants

**Figure 3:**
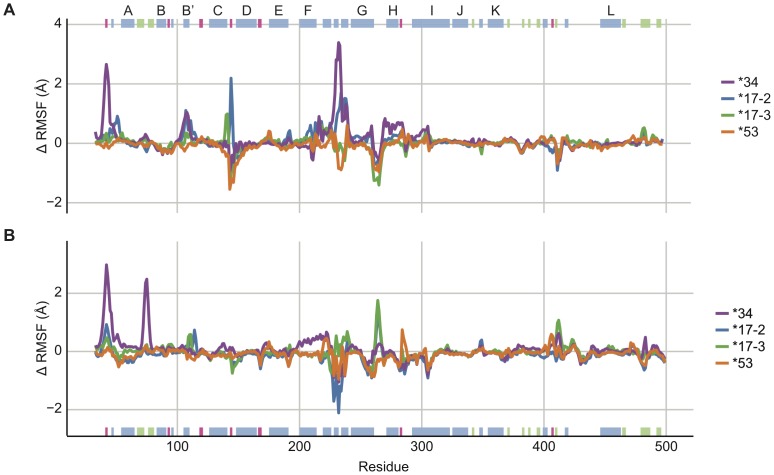
Changes in root mean square fluctuations (ΔRMSF) of backbone atoms of *34 (purple), *17-2 (blue), *17-3 (green), and *53 (orange) relative to *1 (corresponds to zero line) with (A) no ligand bound and (B) with SCH 66712 bound. Positive values for ΔRMSF correlate to atoms of increased flexibility while negative values for ΔRMSF correlate with more rigid atoms compared to reference CYP2D6*1. The x-axis indicates amino acid position. Helices are indicated in blue (and labeled at the top of Panel A), β-sheets in green, and turns in pink. The F-G, C-D, and G-H loop regions show the greatest variability in flexibility between variants and *1 both with and without SCH 66712 bound. There was also a modest difference in flexibility in the meander loop region.

Fluctuation of the backbone atoms was examined by measurement of changes in root mean square fluctuation (ΔRMSF) relative to *1 (Figure 3A). In general, the structural core (helices D, E, I, J, K, L) of all the variants were of similar rigidity as the *1 model.

For the *34, *17-2, and *17-3 collection of sequentially increasing mutations, the greatest differences in structural conformations in the absence of ligand were in the C-D, F-G, and G-H loop regions and helix H (Figures 3A and Figure S4). The F-G loop region of *34 was particularly more flexible than *1 and the other variants. *17-2 was also flexible in the F-G loop region, though not as much as *34; *17-3 was slightly more rigid than *1 in the same region. However, only *17-2 and *17-3 showed increased flexibility in the C-D loop region. At helix H, *34 showed the greatest flexibility followed by *17-2 and *17-3. *53 was generally more rigid than *1 and the other variants, particularly in the F-G loop region and also the C-D loop region. The meander loop (between helices K′ and L) was slightly more rigid in all the variants than in *1 in the absence of ligand.

Fluctuations on the N- and C-terminal ends were not considered since the ends are on the surface of the structure and have no regular secondary structure features allowing greater flexibility.

### Docking of SCH 66712

**Figure 4:**
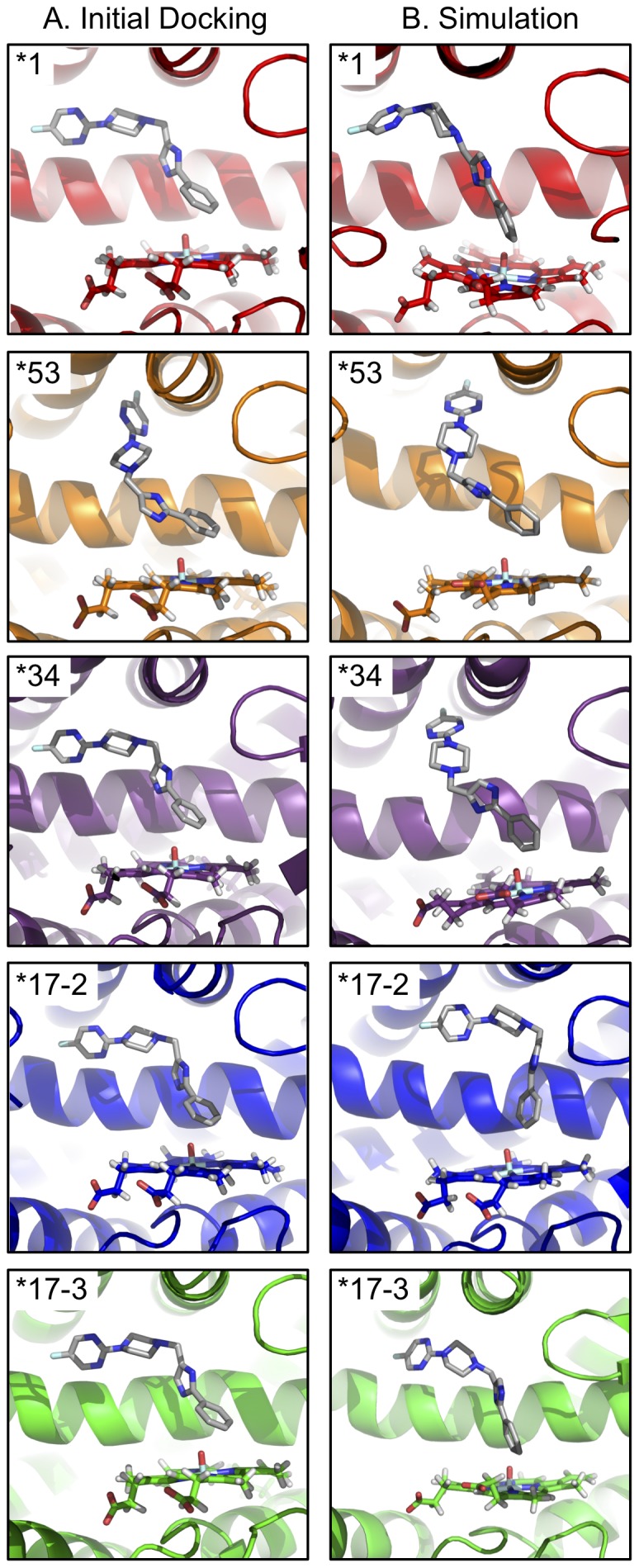
Interaction of SCH 66712 with allelic variants. (A) Initial docking poses of SCH 66712 with each CYP2D6 variant. SCH 66712 was docked using AutoDock Vina as described in the Methods. Other poses not shown include positioning of the heteroaromatic ring above the heme iron. Both positions, phenyl or heteroaromatic ring pointing toward heme, are consistent with known sites of metabolism by *1 [21]. (B) Representative snapshots of the most prominent SCH 66712 binding mode for each variant as determined by PCA from 1000 snapshots (PCA population kernels shown in Figure S12). Additional, minor, binding modes were also identified for *1 and *17-3 as indicated in Figure S13. Helix I is shown in the background. For each variant, the phenyl ring of SCH 66712 points toward the heme. In *1, *17-2, and *17-3 the heteroaromatic ring of SCH 66712 is pointing to a pocket between helices I and G. In *53 and *34, SCH 66712 is oriented with the heteroaromatic ring pointing to a pocket between helices F and E.

SCH 66712 was docked into each variant by use of Autodock Vina as described in the Methods. The lowest energy binding modes with the phenyl group of SCH 66712 oriented toward the heme were selected as starting points for simulations described below since our previous studies suggested that the phenyl group on the substituted imidazole is involved in protein adduction during mechanism-based inactivation [21]. Initial poses for SCH 66712 binding are shown in Figure 4A.

### Molecular Dynamics Simulations of CYP2D6 Variants Bound to SCH 66712

Molecular dynamics simulations were run starting with CYP2D6 variants bound to SCH 66712. As in no-ligand bound simulations, the systems converged at ∼30 ns, except *34 that remained more dynamic (Figure S5). The fluctuations of the backbone atoms were examined by measurement RMSF. To compare changes in simulations with ligand bound, the variants were compared to *1 with ligand bound (Figure 3B and Figure S7). As in simulations without ligand bound (Figure 3A), the core structural elements were largely of similar rigidity as *1 (helices D, E, I, J, K, L). In contrast, with ligand bound all the variants had a moderately more flexible meander loop than *1, and the C-D and F-G loops were more rigid. The G-H loop region was increasingly flexible in the *34, *17-2, and *17-3 series, with *17-3 the most flexible and *34 the least. The F-G loop region showed similar and greater rigidity for all three variants as compared to *1. There was also more rigidity in the H-I turn region for all three. Only *34 showed higher flexibility in the β1-1 and β1-2 sheets when ligand was bound. For *53, the differences from *1 closely matched those in the no-ligand bound simulation except for the F-G and meander loop that were slightly more flexible than *1 in the presence of ligand.

Comparisons for each variant with and without ligand bound were also made (Figures S7, S8, S9, S10, and S11). *1 simulations with ligand bound showed decreases in flexibility in the G-H and C-D loop regions upon ligand binding (Figure S7). Smaller differences in flexibility were measured in helix I and the meander loop regions where the presence of ligand increased flexibility in *1. For *34, ligand simulations were highly more rigid in the F-G loop region and to a lesser extent in the B′-C turn region (Figure S8). While *17-2 displayed the same decrease in flexibility in the F-G loop in the ligand bound form, there was also additional rigidity in the C-D turn region in the ligand bound simulations that was not present in *34 simulations (Figure S9). *17-3, like *17-2, was more rigid in the C-D turn region in ligand bound simulations; however, unlike the others in the series, *17-3 showed increased flexibility especially in the G-H loop region and also in the meander loop region in simulations with ligand (Figure S10). The *53 variant showed slightly more flexibility in the helix G′ region (around residue 230) in ligand bound simulations. The rigidity of the C-D loop region in simulations without ligand was similar in simulations with ligand bound for *53. Overall, *53 showed little conformational differences between no ligand and ligand bound simulations and tended to be the most rigid of the structures (Figure S11).

**Figure 5:**
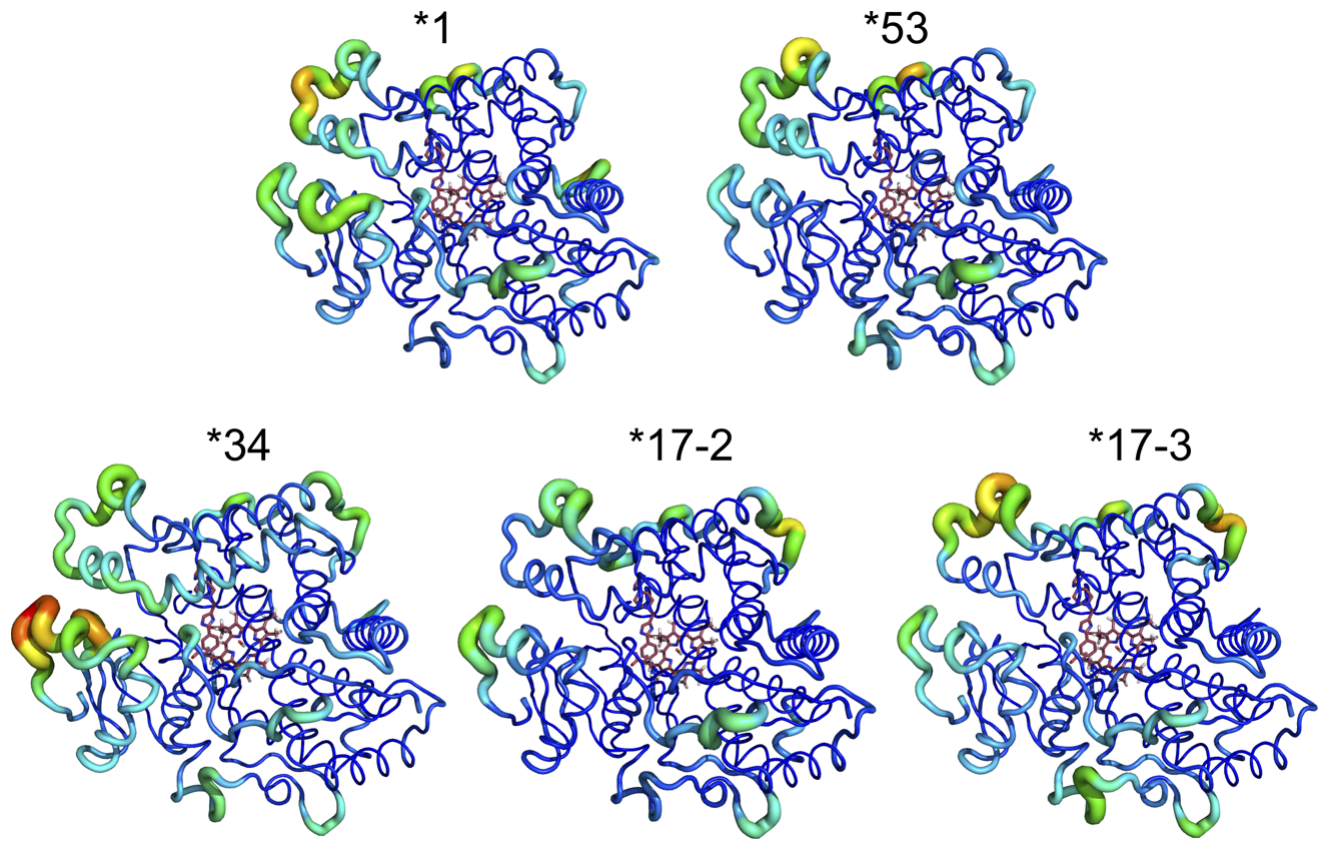
Structural fluctuations of each CYP2D6 variant with SCH 66712 bound. Rainbow color scheme indicates degree of fluctuation with blue indicating little fluctuation to red indicating large fluctuation. The size of the backbone strand is also indicative of fluctuation with large diameter indicative of fluctuations. All structures showed a rigid core surrounding the heme with the area of the greatest flexibility in the F-G loop and helix A′ regions on the distal side. *34 was the most flexible of the variants and *53 was the most rigid.

Differences in flexibility of each variant when bound to SCH 66712 are also visualized by use of putty models made with RMSF values (Figure 5). *53 is the most rigid of the variants. To analyze more specific areas of difference, each structure was overlaid with *1 (Figure S6). The RMSD for the overlays ranged from 1.389 Å to 1.655 Å and in all cases more changes were noted on the distal side than on the proximal side of the structures. For *17-2, the G helix is two amino acids shorter on the C-terminal end, the helix G″ is displaced, and the helix A′ did not form; however, the distance between helix F′ and the region of helix A′ was the same width, ∼10 Å (Figure S6). In *17-3, helix G″ did not form, helix A′ is two amino acids shorter, helix F′ is displaced into the channel narrowing it to ∼7 Å (Figure S6). Comparison of *1 with *34 shows a wider channel near the F-G loop (∼19 Å in *34) as well as a slightly displaced helix G″ and shorter helix A′. Beta sheets 1-1 and 1-2 are also each shorter by two amino acids. Finally, in the overlay of *1 with *53 it is apparent that helix G″ and helix A′ are not formed. Analysis of amino acid positioning in the active site showed only small displacements (typically <1.5 Å) (data not shown).

### Ligand Fluctuations

**Figure 6:**
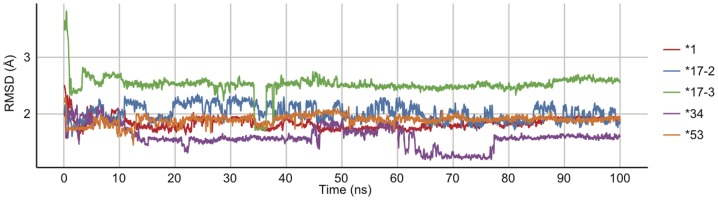
Root square mean deviation of SCH 66712 within active site of each CYP2D6 variant shows system convergence (each variant is colored as indicated in **Figure 3** **).** *53 and *1 reach equilibrium within 20 ns, *17-2 and *17-3 within 50 ns, and *34 does not converge after 100 ns.

To understand how the ligand motions varied in the active site of each variant, fluctuations of SCH 66712 over time were measured in molecular dynamics simulations with SCH 66712 docked. The initial docking pose was as described above with the phenyl group of SCH 66712 pointing towards the heme. RMSD were calculated during the simulation based on initial coordinates for SCH 66712 (Figure 6).

The most stable SCH 66712 motion was seen in *53 that reached RMSD equilibrium in ∼12 ns, just before *1 (∼15 ns). RMSD equilibration with *17-2 and *17-3 occurred later (between 50–80 ns) (Figure 6). Variant *34 did not converge within the 100 ns of the simulation. Binding orientation of SCH 66712 (with phenyl group towards the heme) remained the same at the end of the simulations with ligand close to the ferryl oxygen. The initial spike in the RMSD of *17-3 is due to the meander loop extending away and then retracting toward the core (Figure 6 and data not shown). Overall, *17-3 has the most mutations of the variants studied and it is possible that these mutations affect the stability of the overall structure, as might be suggested by the RMSD.

The most prominent motions of SCH 66712 in the active site of each of the variants during stable simulations were characterized using principal component analysis (PCA) to illustrate binding modes (Figure S12). Application of PCA to *1 yielded one dominant population for SCH 66712 binding and one minor (Figure 4B and Figures S12 and S13). For *34, *17-2, and *53 series, one major population was seen (Figure 4B and Figure S12). However, three distinct populations were noted for *17-3 (one major and two minor) (Figure 4B and Figures S12 and S13).

Distances from the phenyl ring of SCH 66712 to the heme coordinated oxygen were ∼2-5 Å consistent with metabolism and other docking distances for CYP2D6 substrates [45]. SCH 66712 binding energies were calculated using molecular mechanics-Poisson Boltzmann surface area (MM-PBSA) and ranged from −9.25 to −13.05 kcal/mol (Table 2). *53 showed the lowest binding free energy and thus highest affinity for SCH 66712 of the variants (−13.05±2.80 kcal/mol). Our group has previously measured the free energy of SCH 66712 binding to *1 (−9.10±2.33 kcal/mol) and it is in agreement with the value calculated from our simulated model (−9.59±3.84 kcal/mol) (Table 2) [22].

**Table 2:** Total free energy of SCH 66712 binding estimated by MM-PBSA.

	*1	*17-2	*17-3	*34	*53
ΔG_calc_ (kcal/mol)	−9.59(3.84)^1^	−9.25(3.08)	−11.76(2.97)	−10.62(3.29)	−13.05(2.80)
ΔG_exp_ 2 (kcal/mol)	−9.10(2.33)	-	-	-	-

1 Corresponding errors for each value are shown in parenthesis.

2 ΔG_exp_ was calculated using spectral binding constants, *K*_s_, and the relationship: ΔG_exp_ = RT ln *K*_s_, as previously reported [22].

### Analysis of Ligand Channels

Crystal structures of CYP2D6 include a no ligand bound form (2F9Q) [18] and reversible inhibitor bound forms (3QM4 with prinomastat and 3TDA and 3TBG soaked replacement with thioridazine) [23]. These static models show a difference in active site cavity size and shape as well as access channels (reviewed in [10]). Molecular dynamics allows a larger range of structural analysis of putative ligand channels to be performed. Previous studies have shown that ligand access to P450 active sites varies with structural dynamics of the enzyme [44]. In order to understand the role of substrate access and egress in the allelic variants as compared to *1, we analyzed the presence, prominence, and size of channels in each of our variants over the course of our simulations.

**Figure 7:**
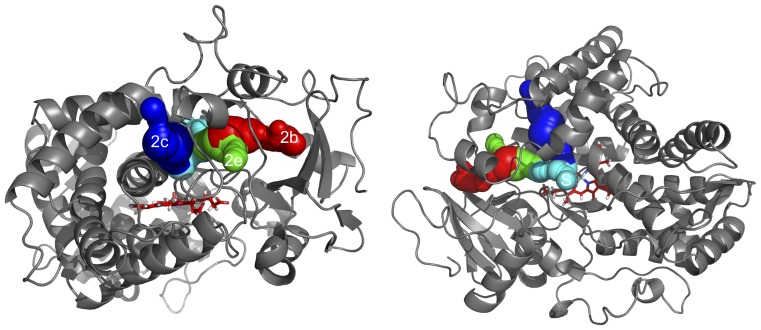
Major tunnels identified in CYP2D6 throughout the MD simulations all depicted in one frame and shown from two views. Tunnels are shown using van der Waals representations. Pathways were determined from 1000 snapshots from the molecular dynamics simulations using the PyMOL plugin CAVER 3.0 and are depicted together on frame 500 of *1. The channels shown are 2c (blue), 2e (green), 2b (red), and Solvent (turquoise). Channel names are given using the nomenclature of Cojocaru et al. [46]. Heme is shown in red sticks. For clarity, other channels identified are not shown. Channel 2c exits between helix I and the B-C loop; channel 2e exits between the B-C and B-B′ loops; channel 2b exits between the B-B′ loop and β-1/β-3 sheets; and the solvent channel exits between helices I and F and sheet β-4.

The highest ranked pathways in the allelic variants were subclasses of channel 2 that are common among CYPs [46], [47]. The top ranked channels for each variant were: 2c in *1, 2f in *17-2, 2a in *17-3, and 2b in both *34 and *53. Collectively, each channel had different rates of occurrence in the models, but all variants contained channel 2b and 2e as major pathways (e.g. in top four of measured clusters). Both channel 2b and 2e open near the B-B′ loop with 2b opening on the side near β-sheet and 2e in the middle of the B-C loop region (Figure 7). The solvent channel and channel 2c were also among the top ranked pathways with 2c opening between the B-C loop and helix I and the solvent channel between helices F and I (Figure 7). All of the subclass 2 channels are near sites of mutations in the allelic variants in this study: position 296 is at the start of helix I (near channel 2c), position 107 is in helix B′ (near channels 2b and 2e), and positions 120 and 122 are both in the B-C loop and SRS1 (near start point for essentially all subclass 2 channels, particularly channels 2b and 2e, due to proximity to the heme center). Position 486 is in sheet β4-2 and potentially near the solvent channel.

**Figure 8:**
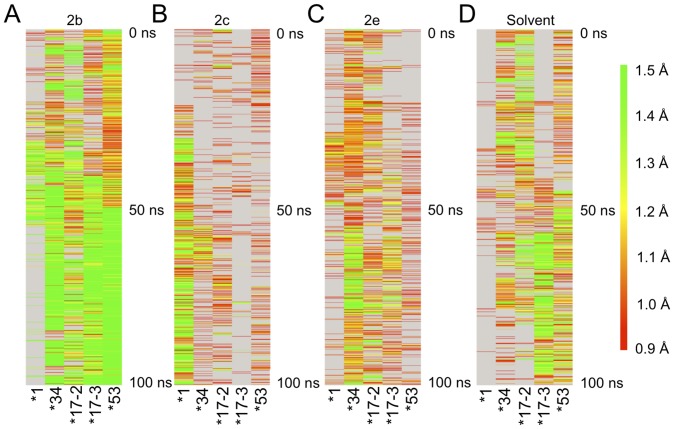
Time evolution of the bottleneck radius of channels 2b, 2c, 2e, and solvent in each CYP2D6 variant during simulations. For analysis, 1000 snapshots of each variant were used. The snapshots were taken each 100 ps over a 100 ns simulation time. The color map ranges from 0.9 Å (red) to 1.5 Å or greater (green) bottlenecks. Grey indicates that no tunnel with bottleneck radius ≥0.9 Å was identified for the given pathway cluster. Channel 2b was the most open channel for the variants, but not *1. Furthermore, over the course of the simulation, 2b became more open for the variants. Channel 2c was the major channel for *1, but was not open frequently or wide for any of the variants. Channel 2e was most open in *34. The solvent channel was a major open channel for the variants, particularly *17-3, but was only open a few times for *1 over the course of the 100 ns simulation. CAVER 3.0 was used for channel identification as described in the methods.

Comparison of the time evolution of the 2b channels among variants showed the most open conformations in terms of width and duration in *53 followed by *34 (Figure 8A). *17-2 and *17-3 were similar and also more open and for longer time than *1 (Figure 8A). Channel 2c was the dominant channel in *1, but not ranked in the top ten clusters for *17-2 and *17-3 nor in top five clusters for *34 or *53 as shown by the narrow opening and short open times for the variants as compared to *1 (Figure 8B). The 2e channel was most open in the *34 as compared to the other variants (Figure 8C). The solvent channel was not a dominant channel in *1, but was in the other variants, particularly the *17-2 and *17-3 polymorphisms (Figure 8D).

## Discussion

We have previously shown SCH 66712 to be a potent inactivator of CYP2D6 *1 [21]. However, the ability of SCH 66712 to interact with and inactivate polymorphic forms of CYP2D6 is unknown. In the current study, molecular dynamics simulations of four allelic variants of CYP2D6 showed that SCH 66712 docked to the active site of each variant, though there were differences in flexible regions of each variant that might affect substrate binding and product release (Figure 3). Binding orientations were consistent with metabolism and possible protein adduction (Figure 4B) and calculated binding energies also were consistent with favorable binding (Table 2). Analysis of channel formation over time further indicate that SCH 66712 would have access to the active sites of each variant, though predominant pathways of access/egress varied (Figure 8).

While the global structural fold for each variant was the same as the *1 model and the 3MQ4 crystal structure, differences in flexibility of local regions were observed (Figure 3 and Figures S2 and S3). Specifically, more flexible F-G and C-D loop regions and a more rigid G-H loop region in comparison to *1 for *34, *17-2, and *17-3 were observed when no SCH 66712 was bound. Upon simulations with ligand bound at the active site, the flexibility of the F-G loop region decreased while the flexibility of the G-H loop increased (Figure 3). These computational observations suggest that reduced activity of *34, *17-2, and *17-3 may be due to changes in regional structural flexibility. Protein flexibility alone as a major determinant of varied enzyme activity for polymorphic forms of CYP2D6 is also consistent with computational analysis of catalyzed reactions. That is, the Arrhenius equation relates rates of reactions to reaction temperature and reaction energy. A modified view of the Arrhenius equation as it applies to enzyme catalyzed reactions shows:
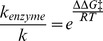
(Equation1)where *k*_enzyme_ is the rate of an enzymatic reaction, *k* is the non-enzyme catalyzed rate, *e* is Euler's number, ΔΔG^‡^ is the activation energy difference between the enzymatic and non-enzymatic reactions, R is the gas constant, and T is temperature. For a reaction that displays no more than a 5-fold difference in reaction rate, such as for the allelic variants in this study compared to *1, the ΔΔG^‡^ is only ∼3.7 kJ/mol, or the strength of a single hydrogen bond, van der Waals interaction, or London dispersion force. From this, it is expected that only small differences in protein structure in the polymorphisms would be observed, as noted in this study. Thus, changes in enzyme activity might be better explained by conformational dynamics and changes in active site accessibility particularly since all variants (save Phe120Ile in *53) occur distal to the active site.

Other groups have found that CYP structural rigidity/flexibility is affected by ligand binding [23], [45], [48], [49], [50], [51] and that the F-G loop region influences interaction with substrates [23], [44] as also shown in the simulations in the present study. Fluctuations of the F-G loop have previously been identified as the source of the largest movements during simulations for many CYPs [47]. The F-G loop region also borders many of the substrate egress/access channels. Furthermore, flexibility is important for substrate access and specificity [46], [48], [51], [52], [53], [54].

Mutations introduced along substrate access channels of CYP2B6 and CYP2B4 increased *K*_m_ values and lowered enzyme efficiency [52]. Zhang et al. found previously with a single distal mutation at F186 of CYP1A2 that altered enzyme activity might be explained by “access mechanism” with regard to channel size and opening over time [15]. Also, for the F186 mutation of CYP1A2, greater variation was observed in the D-E helices region as compared to reference CYP1A2. However, in our simulations with CYP2D6, the F-G loop, helix F, C-D loop, and, to a lesser degree, the meander loop were the regions of greatest differences in flexibility in the allelic variants. The meander loop has a role in heme binding and stabilization of the tertiary structure and has been shown previously to have reverse flexibility in ligand-free and ligand-bound structures as also observed in simulations here [17], [44], [55]. These findings show that for different CYPs different flexibility changes will be observed that affect enzyme efficiency. Overall, our molecular dynamics results with and without ligand present are consistent with previous findings by Berka et al. that showed greater flexibility in CYP2D6 in the absence of ligand and greater rigidity upon binding of quinidine ([50], model built from 2F9Q).

Increasing numbers of distal mutations in allelic variants *34, *17-2, and *17-3 did not render significantly different global protein conformation or structural effects on the active site. This is consistent with the notion that distal mutations may act to change protein flexibility/rigidity [12], [15]. The effects observed here, including greater flexibility with no ligand bound in the F-G loop region and greater rigidity in the same region upon ligand binding, suggests that differences in enzyme activity between the *34, *17-2, and *17-3 (distal allelic variants) may be caused by an “access mechanism” with regard to channels and substrate access to the active site (terminology of Zhang et al. [15]). Furthermore, the observations of few global effects with the *53 (close to active site allelic variant) also supports the idea that differences in flexibility/rigidity can be major determinants of enzyme activity since *53 is overall more rigid than *1 both with and without ligand bound.

Access to the active site via substrate channels may be altered by changes in flexibility of the enzyme. Analysis of substrate channels revealed that there are differences in dominant pathways as well as size and duration of openings. Most previous analysis of substrate access and egress channels in CYP2D6 have used the no-ligand bound 2F9Q structure for analysis with or without molecular dynamics simulations [46], [47], [50], [56]. Our analysis with 3QM4 and molecular dynamics is one of the first to look at channel evolution in the “closed” structure of CYP2D6. CAVER analysis found channels 2b, 2e, and 2c to be the predominant channels in all the structures. However, the solvent channel was not a major channel for *1. Previously, the solvent channel in CYP2D6 was the only channel identified in analysis of the static crystal structure of CYP2D6 (2F9Q) and it was thought that the solvent channel might also be a site for substrate access [18], [46], [56]. In comparison to 2F9Q, analysis of the static prinomastat structure of CYP2D6 (3QM4) showed a more open channel 2b; likewise the thioridazine bound CYP2D6 (3TBG) showed more open, and merged, channels 2a, 2b, and 2f [10], [23]. In our *1 model from molecular dynamics, the roles of channels other than solvent also appeared more likely since the solvent channel was not a major access channel for *1. Tunnel analysis of CYP3A4 simulations by others also did not show solvent channel as a major access pathway [44]. However, the solvent channel was a major pathway in the variants such that alterations in interactions with substrates and inactivators could be reflected in different principle modes of access to and from the active site. In a series of CYPs (2A6, 2D6, 2B4, 2E1, 2C9, 3A4) studied by molecular dynamics methods, the widest channels were shown to typically be 2b, 2e, and solvent [47]. Further, access channel bottlenecks fluctuated around 1.5 Å, too small for ligand passage, as we observed in our analysis [47]. To compensate for small channels, Hendrychova et al. suggested that substrates may induce conformational changes as they enter the access channels and this may be related to the presence of the SRS along the channels. Also, the time evolution of channels showed that channels were generally open more and with greater radii the longer the simulations ran (Figure 8; exception of *1 and channel 2b). Since 100 ns is less than expected *in vivo* time scales for protein dynamics (µs), it is possible that channels would open to a greater extent over longer time [48]. The combination of these findings highlight the importance of structural flexibility that can be examined in molecular dynamics simulations.

The binding orientations of SCH 66712 in all variants were consistent and imply that similar interactions of the inactivator with the heme catalytic center should occur during catalysis. Since a key aspect of mechanism-based inactivation is progression through the P450 catalytic cycle at the heme in the active site, the allelic variants would likely be as susceptible to mechanism-based inactivation as seen with *1, though binding may be slowed or altered in the variants due to changes in substrate recognition sites and distance from the heme. Handa et al. previously showed for MD models of *1 and *17-3 that when docking positions for reversible inhibitors (fluoxetine and norfluoxetine, reversible inhibitors) were similar, so were inhibition kinetics measured by *K*_i_ [41]. Conversely, when docking orientations were different, so were *K*_i_ values and inhibitory activity (e.g. cocaine, imipramine, quinidine, and thioridazine) [41]. Since SCH 66712 binds in similar orientations at similar distances between the heme iron and the phenyl ring of SCH 66712 for all the variants in this study, we predict that these variants will be susceptible to inactivation by SCH 66712 in similar way to *1. However, the inactivation may be altered in *53 due to lower calculated binding free energy, greater rigidity in structure, and variability in substrate access channels observed in our computations.

*53 has previously been reported to be a possible UM of CYP2D6 substrates and has two mutations: F120I and A122S [6]. F120 has a role in substrate binding and in particular is thought to interact with and orient aromatic groups of CYP2D6 substrates. However, alignment of the B-C loop region of CYP2 subfamily members from various species shows that position 120 is as likely to be an F, V, or I [57]. Thus, the F120I mutation may not have a large effect on substrate binding in *53. In an overlay of *1 and *53, F and I at position 120 occupy the same space (data not shown). The A122S mutation is a change in polarity for position 122 and SRS1 of the B-C loop, though no new hydrogen bonds between protein residues or backbone are formed as a result of the change. The backbone carbonyl at position 122 is hydrogen bonded to R441 in both *1 and *53 (data not shown). However, this does not rule out the possibility that interactions with putative substrates as they access the active site are changed due to the change in polarity.

Simulations of *53 support a designation of UM. Unlike simulations with *17-2, *17-3, and *34, that are lower activity CYP2D6 variants, the *53 variant showed highly stable interaction with SCH 66712 and reached stable plateaus faster than the other variants and *1 (Figure S2 and S5). Others have noted that CYPs with faster equilibration times in molecular dynamics also were physically more stable *in vitro* [47]. The structural basis for the stability of *53 over the other variants is seen globally in greater rigidity in the core, proximal, and distal parts of the structure. Differences in flexibility in the allelic variants may give rise to the observed differences in substrate metabolism and selectivity since enzyme flexibility is important for specificity [47], [48], [54].

## Conclusions

SCH 66712 interacts with allelic variants *34, *17-2, *17-3, and *53 similar to interactions with *1. Binding orientations and energies, access to the active site, and stability of protein in presence of SCH 66712 indicate that these polymorphic forms would be susceptible to inactivation in ways similar to *1. In addition to understanding interaction of SCH 66712 with variants of CYP2D6, by examining four allelic variants in parallel our study gives a broader perspective on what can be expected in molecular dynamics simulations of polymorphic forms of CYPs. That is, both distal mutations and those near the active site produced the same structural fold as the original crystal structure but the fold is coupled to local changes in protein flexibility and to substrate access channel openings and predominant pathways. Furthermore, UM phenotypes such as *53 may be due to increased rigidity of protein structure.

## Supporting Information

Figure S1 Root mean square fluctuations (RMSF) of backbone atoms of *1 with SCH 66712 bound for three separate simulations. Fluctuations across the three simulations were similar. Simulation 5 was used for comparison to allelic variants in Figure 3. Helix nomenclature is indicated at top.
(TIFF)

Figure S2 Root mean square deviations of backbone atoms of CYP2D6*1, *34, *17-2, *17-3, and *53 with no ligand bound. RMSD of all backbone atoms converged at 1.7 to 2.3 Å.
(TIFF)

Figure S3 Superposition of 3QM4 (cyano) and simulation model CYP2D6*1 (red). (A) Regions of structural difference include the appearance of helix A′ in the model, displacement of helix F′ and the F-G loop, and shortening of helix G′. Channel 2b/2a/2f is indicated by an arrow. (B) Slab view of ligand binding shows that prinomastat, with a nitrogen, is coordinated directly to the heme iron in 3QM4 while SCH 66712 in the CYP2D6*1 model is not coordinated directly to the heme (RMSD 1.724 Å). Other moieties of the two ligands overlay in the active site.
(TIFF)

Figure S4 Superposition of *34, *17-2, and *17-3 from simulations. Areas of greatest difference are indicated with arrows and include the helix G″ (helix G″ is not formed in *17-3), the helix B′, the helix F′, and the F-G loop regions. The RMSD for the superposition was less than 2 Å.
(TIFF)

Figure S5 Root mean square deviations of backbone atoms of CYP2D6*1, *17-2, *17-3, *34, and *53 with the ligand SCH 66712 bound. RMSD of all backbone atoms converged at 1.5 to 2.1 Å except for *34.
(TIFF)

Figure S6 The *1 model was superimposed on each of the variants with SCH 66712 bound. (A) *1 with *34 (RMSD 1.551 Å); (B) *1 with *17-2 (RMSD 1.655 Å); (C) *1 with *17-3 (RMSD 1.389 Å); and (D) *1 with *53 (RMSD 1.525 Å). Areas of greatest difference were on the distal side of the protein, particularly the F-G loop, helix F, and helix G″ regions.
(TIFF)

Figure S7 RMSF with and without ligand bound for *1. The G-H loop region is more flexible in the absence of ligand. Other regions display similar rigidity with our without ligand bound. RMSF with no ligand is shown in red. RMSF with SCH 66712 bound is shown in grey. Regions of helices are shown in blue, β-sheets in green, and turns in pink. Helix nomenclature is indicated at top.
(TIFF)

Figure S8 RMSF with and without ligand bound for *34. The F-G loop region is more flexible in the absence of ligand. Other regions display similar rigidity with our without ligand bound. RMSF with no ligand is shown in purple. RMSF with SCH 66712 bound is shown in grey. Regions of helices are shown in blue, β-sheets in green, and turns in pink. Helix nomenclature is indicated at top.
(TIFF)

Figure S9 RMSF with and without ligand bound for *17-2. The F-G and C-D loop regions are more flexible in the absence of ligand while most other areas have similar flexibility with or without ligand. RMSF with no ligand is shown in blue. RMSF with SCH 66712 bound is shown in grey. Regions of helices are shown in blue, β-sheets in green, and turns in pink. Helix nomenclature is indicated at top.
(TIFF)

Figure S10 RMSF with and without ligand bound for *17-3. In the absence of ligand, the G-H loop is more rigid while the C-D loop is more flexible. The meander loop is also more flexible with ligand bound. RMSF with no ligand is shown in green. RMSF with SCH 66712 bound is shown in grey. Regions of helices are shown in blue, β-sheets in green, and turns in pink. Helix nomenclature is indicated at top.
(TIFF)

Figure S11 RMSF with and without ligand bound for *53. RMSF were similar with and without ligand bound. Small differences in fluctuations were observed in the F-G and G-H loop regions with greater flexibility when ligand was bound. RMSF with no ligand is shown in orange. RMSF with SCH 66712 bound is shown in grey. Regions of helices are shown in blue, β-sheets in green, and turns in pink. Helix nomenclature is indicated at top.
(TIFF)

Figure S12 Principal component analysis for SCH 66712 binding to each CYP2D6 variant: (A) CYP2D6*1, (B) CYP2D6*53, (C) CYP2D6*34, (D) CYP2D6*17-2, (E) CYP2D6*17-3. Plots are shown as kernel densities with black dots indicating the time series for the PCA; the transparency of the dots is a function of time providing emphasis on the later time points (darker). The green dot in each plot represents the location of the PDB snapshot shown in Figure 4B as representative of the binding patterns for each isoform.
(TIFF)

Figure S13 Representative snapshots of the minor binding modes from PCA in Figure S12 for SCH 66712 in (A) *1 and (B) *17-3. Helix I is shown in background. In all poses the phenyl group of SCH 66712 is pointing toward the heme with the heteroaromatic group oriented toward helix G.
(TIFF)

Scheme S1 **C programming language script used for identification of the coordinates of the oxygen and iron of Compound I and for defining the starting point for CAVER analysis.**
(DOCX)

## References

[1] Guengerich FP (2003) Cytochromes P450, drugs, and diseases. Mol Interv 3: 194–204. 14993447 10.1124/mi.3.4.194

[2] Wienkers LC, Heath TG (2005) Predicting in vivo drug interactions from in vitro drug discovery data. Nat Rev Drug Discov 4: 825–833. 16224454 10.1038/nrd1851

[3] Wang B, Yang LP, Zhang XZ, Huang SQ, Bartlam M, et al (2009) New insights into the structural characteristics and functional relevance of the human cytochrome P450 2D6 enzyme. Drug Metab Rev 41: 573–643. 19645588 10.1080/03602530903118729

[4] Ingelman-Sundberg M (2005) Genetic polymorphisms of cytochrome P450 2D6 (CYP2D6): clinical consequences, evolutionary aspects and functional diversity. Pharmacogenomics J 5: 6–13. 15492763 10.1038/sj.tpj.6500285

[5] Niwa T, Murayama N, Yamazaki H (2011) Comparison of cytochrome P450 2D6 and variants in terms of drug oxidation rates and substrate inhibition. Curr Drug Metab 12: 412–435. 21453270 10.2174/138920011795495286

[6] Sakuyama K, Sasaki T, Ujiie S, Obata K, Mizugaki M, et al (2008) Functional characterization of 17 CYP2D6 allelic variants (CYP2D6.2, 10, 14A-B, 18, 27, 36, 39, 47-51, 53-55, and 57). Drug Metab Dispos 36: 2460–2467. 18784265 10.1124/dmd.108.023242

[7] Zhou SF (2009) Polymorphism of human cytochrome P450 2D6 and its clinical significance: Part I. Clin Pharmacokinet. 48: 689–723. 10.2165/11318030-000000000-00000 19817501

[8] Zhou SF (2009) Polymorphism of human cytochrome P450 2D6 and its clinical significance: part II. Clin Pharmacokinet 48: 761–804. 19902987 10.2165/11318070-000000000-00000

[9] Hollenberg PF, Kent UM, Bumpus NN (2008) Mechanism-based inactivation of human cytochromes p450s: experimental characterization, reactive intermediates, and clinical implications. Chem Res Toxicol 21: 189–205. 18052110 10.1021/tx7002504

[10] Johnson EF, Stout CD (2013) Structural diversity of eukaryotic membrane cytochrome p450s. J Biol Chem 288: 17082–17090. 23632020 10.1074/jbc.R113.452805 PMC3682514

[11] Robarge JD, Li L, Desta Z, Nguyen A, Flockhart DA (2007) The star-allele nomenclature: retooling for translational genomics. Clin Pharmacol Ther 82: 244–248. 17700589 10.1038/sj.clpt.6100284

[12] Lertkiatmongkol P, Assawamakin A, White G, Chopra G, Rongnoparut P, et al (2013) Distal effect of amino acid substitutions in CYP2C9 polymorphic variants causes differences in interatomic interactions against (S)-warfarin. PLoS One 8: e74053. 24023924 10.1371/journal.pone.0074053 PMC3759441

[13] Sano E, Li W, Yuki H, Liu X, Furihata T, et al (2010) Mechanism of the decrease in catalytic activity of human cytochrome P450 2C9 polymorphic variants investigated by computational analysis. J Comput Chem 31: 2746–2758. 20839301 10.1002/jcc.21568

[14] Cui YL, Zheng QC, Zhang JL, Xue Q, Wang Y, et al (2013) Molecular dynamic investigations of the mutational effects on structural characteristics and tunnel geometry in CYP17A1. J Chem Inf Model 53: 3308–3317. 24205838 10.1021/ci400553w

[15] Zhang T, Liu LA, Lewis DF, Wei DQ (2011) Long-range effects of a peripheral mutation on the enzymatic activity of cytochrome P450 1A2. J Chem Inf Model 51: 1336–1346. 21598960 10.1021/ci200112b

[16] Oscarson M, Hidestrand M, Johansson I, Ingelman-Sundberg M (1997) A combination of mutations in the CYP2D6*17 (CYP2D6Z) allele causes alterations in enzyme function. Mol Pharmacol 52: 1034–1040. 9415713 10.1124/mol.52.6.1034

[17] Sirim D, Widmann M, Wagner F, Pleiss J (2010) Prediction and analysis of the modular structure of cytochrome P450 monooxygenases. BMC Struct Biol 10: 34. 20950472 10.1186/1472-6807-10-34 PMC3224734

[18] Rowland P, Blaney FE, Smyth MG, Jones JJ, Leydon VR, et al (2006) Crystal structure of human cytochrome P450 2D6. J Biol Chem 281: 7614–7622. 16352597 10.1074/jbc.M511232200

[19] Sridar C, Snider NT, Hollenberg PF (2011) Anandamide oxidation by wild-type and polymorphically expressed CYP2B6 and CYP2D6. Drug Metab Dispos 39: 782–788. 21289075 10.1124/dmd.110.036707 PMC3082373

[20] Yu A, Kneller BM, Rettie AE, Haining RL (2002) Expression, purification, biochemical characterization, and comparative function of human cytochrome P450 2D6.1, 2D6.2, 2D6.10, and 2D6.17 allelic isoforms. J Pharmacol Exp Ther 303: 1291–1300. 12438554 10.1124/jpet.102.039891

[21] Nagy LD, Mocny CS, Diffenderfer LE, Hsi DJ, Butler BF, et al (2011) Substituted imidazole of 5-fluoro-2-[4-[(2-phenyl-1H-imidazol-5-yl)methyl]-1-piperazinyl]pyrimidine Inactivates cytochrome P450 2D6 by protein adduction. Drug Metab Dispos 39: 974–983. 21422192 10.1124/dmd.110.037630 PMC3100908

[22] Livezey MR, Nagy LD, Diffenderfer LE, Arthur EJ, Hsi DJ, et al (2012) Molecular analysis and modeling of inactivation of human CYP2D6 by four mechanism based inactivators. Drug Metab Lett 6: 7–14. 22372551 10.2174/187231212800229318 PMC4037324

[23] Wang A, Savas U, Hsu MH, Stout CD, Johnson EF (2012) Crystal structure of human cytochrome P450 2D6 with prinomastat bound. J Biol Chem 287: 10834–10843. 22308038 10.1074/jbc.M111.307918 PMC3322812

[24] Pettersen EF, Goddard TD, Huang CC, Couch GS, Greenblatt DM, et al (2004) UCSF Chimera–a visualization system for exploratory research and analysis. J Comput Chem 25: 1605–1612. 15264254 10.1002/jcc.20084

[25] Dolinsky TJ, Nielsen JE, McCammon JA, Baker NA (2004) PDB2PQR: an automated pipeline for the setup of Poisson-Boltzmann electrostatics calculations. Nucleic Acids Res 32: W665–667. 15215472 10.1093/nar/gkh381 PMC441519

[26] Dolinsky TJ, Czodrowski P, Li H, Nielsen JE, Jensen JH, et al (2007) PDB2PQR: expanding and upgrading automated preparation of biomolecular structures for molecular simulations. Nucleic Acids Res 35: W522–525. 17488841 10.1093/nar/gkm276 PMC1933214

[27] Li H, Robertson AD, Jensen JH (2005) Very Fast Empirical Prediction and Interpretation of Protein pKa Values. Proteins 61: 704–721. 16231289 10.1002/prot.20660

[28] Trott O, Olson AJ (2010) AutoDock Vina: improving the speed and accuracy of docking with a new scoring function, efficient optimization, and multithreading. J Comput Chem 31: 455–461. 19499576 10.1002/jcc.21334 PMC3041641

[29] Salomon-Ferrer R, Goetz AW, Poole D, Le Grand S, Walker RC (2013) Routine Microsecond Molecular Dynamics Simulations with AMBER on GPUs. 2. Explicit Solvent Particle Mesh Ewald. J Chem Theory Comput 9: 3878–3888. 26592383 10.1021/ct400314y

[30] Case DA, Babin V, Berryman JT, Betz RM, Cai Q, et al.. (2012) AMBER 12. University of California, San Francisco.

[31] Case DA, Cheatham TE 3rd, Darden T, Gohlke H, Luo R, et al (2005) The Amber biomolecular simulation programs. J Comput Chem 26: 1668–1688. 16200636 10.1002/jcc.20290 PMC1989667

[32] Shahrokh K, Orendt A, Yost GS, Cheatham TE 3rd (2012) Quantum mechanically derived AMBER-compatible heme parameters for various states of the cytochrome P450 catalytic cycle. J Comput Chem 33: 119–133. 21997754 10.1002/jcc.21922 PMC3242737

[33] Vanquelef E, Simon S, Marquant G, Garcia E, Klimerak G, et al (2011) R.E.D. Server: a web service for deriving RESP and ESP charges and building force field libraries for new molecules and molecular fragments. Nucleic Acids Res 39: W511–517. 21609950 10.1093/nar/gkr288 PMC3125739

[34] Darden T, Perera L, Li L, Pedersen L (1999) New tricks for modelers from the crystallography toolkit: the particle mesh Ewald algorithm and its use in nucleic acid simulations. Structure 7: R55–60. 10368306 10.1016/s0969-2126(99)80033-1

[35] Miller BRI, McGee TDJ, Swails JM, Homeyer N, Gohlke H, et al (2012) MMPBSA.py: An Efficient Program for End-State Free Energy Calculations. J Chem Theory Comput 8: 3314–3321. 26605738 10.1021/ct300418h

[36] Hou T, Wang J, Li Y, Wang W (2011) Assessing the performance of the MM/PBSA and MM/GBSA methods. 1. The accuracy of binding free energy calculations based on molecular dynamics simulations. J Chem Inf Model 51: 69–82. 21117705 10.1021/ci100275a PMC3029230

[37] Tan C, Yang L, Luo R (2006) How well does Poisson-Boltzmann implicit solvent agree with explicit solvent? A quantitative analysis. J Phys Chem B 110: 18680–18687. 16970499 10.1021/jp063479b

[38] Zou H, Luo C, Zheng S, Luo X, Zhu W, et al (2007) Molecular insight into the interaction between IFABP and PA by using MM-PBSA and alanine scanning methods. J Phys Chem B 111: 9104–9113. 17602517 10.1021/jp0713763

[39] Fu G, Liu H, Doerksen RJ (2012) Molecular modeling to provide insight into the substrate binding and catalytic mechanism of human biliverdin-IXalpha reductase. J Phys Chem B 116: 9580–9594. 22823425 10.1021/jp301456j PMC3505555

[40] Kar P, Knecht V (2012) Mutation-induced loop opening and energetics for binding of tamiflu to influenza N8 neuraminidase. J Phys Chem B 116: 6137–6149. 22553951 10.1021/jp3022612

[41] Handa K, Nakagome I, Yamaotsu N, Gouda H, Hirono S (2013) In silico study on the inhibitory interaction of drugs with wild-type CYP2D6.1 and the natural variant CYP2D6.17. Drug Metab Pharmacokinet 29: 52–60. 23857029 10.2133/dmpk.dmpk-13-rg-044

[42] Chovancova E, Pavelka A, Benes P, Strnad O, Brezovsky J, et al (2012) CAVER 3.0: a tool for the analysis of transport pathways in dynamic protein structures. PLoS Comput Biol 8: e1002708. 23093919 10.1371/journal.pcbi.1002708 PMC3475669

[43] Lampe JN, Brandman R, Sivaramakrishnan S, de Montellano PR (2010) Two-dimensional NMR and all-atom molecular dynamics of cytochrome P450 CYP119 reveal hidden conformational substates. J Biol Chem 285: 9594–9603. 20097757 10.1074/jbc.M109.087593 PMC2843209

[44] Skopalik J, Anzenbacher P, Otyepka M (2008) Flexibility of human cytochromes P450: molecular dynamics reveals differences between CYPs 3A4, 2C9, and 2A6, which correlate with their substrate preferences. J Phys Chem B 112: 8165–8173. 18598011 10.1021/jp800311c

[45] Hritz J, de Ruiter A, Oostenbrink C (2008) Impact of plasticity and flexibility on docking results for cytochrome P450 2D6: a combined approach of molecular dynamics and ligand docking. J Med Chem 51: 7469–7477. 18998665 10.1021/jm801005m

[46] Cojocaru V, Winn PJ, Wade RC (2007) The ins and outs of cytochrome P450s. Biochim Biophys Acta 1770: 390–401. 16920266 10.1016/j.bbagen.2006.07.005

[47] Hendrychova T, Berka K, Navratilova V, Anzenbacher P, Otyepka M (2012) Dynamics and hydration of the active sites of mammalian cytochromes P450 probed by molecular dynamics simulations. Curr Drug Metab 13: 177–189. 22208532 10.2174/138920012798918408

[48] Otyepka M, Berka K, Anzenbacher P (2012) Is there a relationship between the substrate preferences and structural flexibility of cytochromes P450? Curr Drug Metab 13: 130–142. 22208528 10.2174/138920012798918372

[49] Zhang H, Gay SC, Shah M, Foroozesh M, Liu J, et al (2013) Potent mechanism-based inactivation of cytochrome P450 2B4 by 9-ethynylphenanthrene: implications for allosteric modulation of cytochrome P450 catalysis. Biochemistry 52: 355–364. 23276288 10.1021/bi301567z PMC3568706

[50] Berka K, Anzenbacherova E, Hendrychova T, Lange R, Masek V, et al (2012) Binding of quinidine radically increases the stability and decreases the flexibility of the cytochrome P450 2D6 active site. J Inorg Biochem 110: 46–50. 22459173 10.1016/j.jinorgbio.2012.02.010

[51] Peric-Hassler L, Stjernschantz E, Oostenbrink C, Geerke DP (2013) CYP 2D6 binding affinity predictions using multiple ligand and protein conformations. Int J Mol Sci 14: 24514–24530. 24351831 10.3390/ijms141224514 PMC3876125

[52] Jang HH, Davydov DR, Lee GY, Yun CH, Halpert JR (2014) The role of cytochrome P450 2B6 and 2B4 substrate access channel residues predicted based on crystal structures of the amlodipine complexes. Arch Biochem Biophys 545: 100–107. 24445070 10.1016/j.abb.2014.01.008 PMC4030592

[53] Zhao Y, White MA, Muralidhara BK, Sun L, Halpert JR, et al (2006) Structure of microsomal cytochrome P450 2B4 complexed with the antifungal drug bifonazole: insight into P450 conformational plasticity and membrane interaction. J Biol Chem 281: 5973–5981. 16373351 10.1074/jbc.M511464200

[54] Ekroos M, Sjogren T (2006) Structural basis for ligand promiscuity in cytochrome P450 3A4. Proc Natl Acad Sci U S A 103: 13682–13687. 16954191 10.1073/pnas.0603236103 PMC1564212

[55] Zheng YM, Fisher MB, Yokotani N, Fujii-Kuriyama Y, Rettie AE (1998) Identification of a meander region proline residue critical for heme binding to cytochrome P450: implications for the catalytic function of human CYP4B1. Biochemistry 37: 12847–12851. 9737862 10.1021/bi981280m

[56] Yu X, Cojocaru V, Wade RC (2013) Conformational diversity and ligand tunnels of mammalian cytochrome P450s. Biotechnol Appl Biochem 60: 134–145. 23587001 10.1002/bab.1074

[57] Marechal JD, Kemp CA, Roberts GC, Paine MJ, Wolf CR, et al (2008) Insights into drug metabolism by cytochromes P450 from modelling studies of CYP2D6-drug interactions. Br J Pharmacol 153 Suppl 1: S82–89. 18026129 10.1038/sj.bjp.0707570 PMC2268062

[58] Marez D, Legrand M, Sabbagh N, Lo Guidice JM, Spire C, et al (1997) Polymorphism of the cytochrome P450 CYP2D6 gene in a European population: characterization of 48 mutations and 53 alleles, their frequencies and evolution. Pharmacogenetics 7: 193–202. 9241659 10.1097/00008571-199706000-00004

