# Supplementary figures and images for: Molecular Dynamics of CYP2D6 Polymorphisms in the Absence and Presence of a Mechanism-Based Inactivator Reveals Changes in Local Flexibility and Dominant Substrate Access Channels
Source: PLoS One. 2014 Oct 6;9(10):e108607. doi: 10.1371/journal.pone.0108607 (PMC4186923; doi:10.1371/journal.pone.0108607)

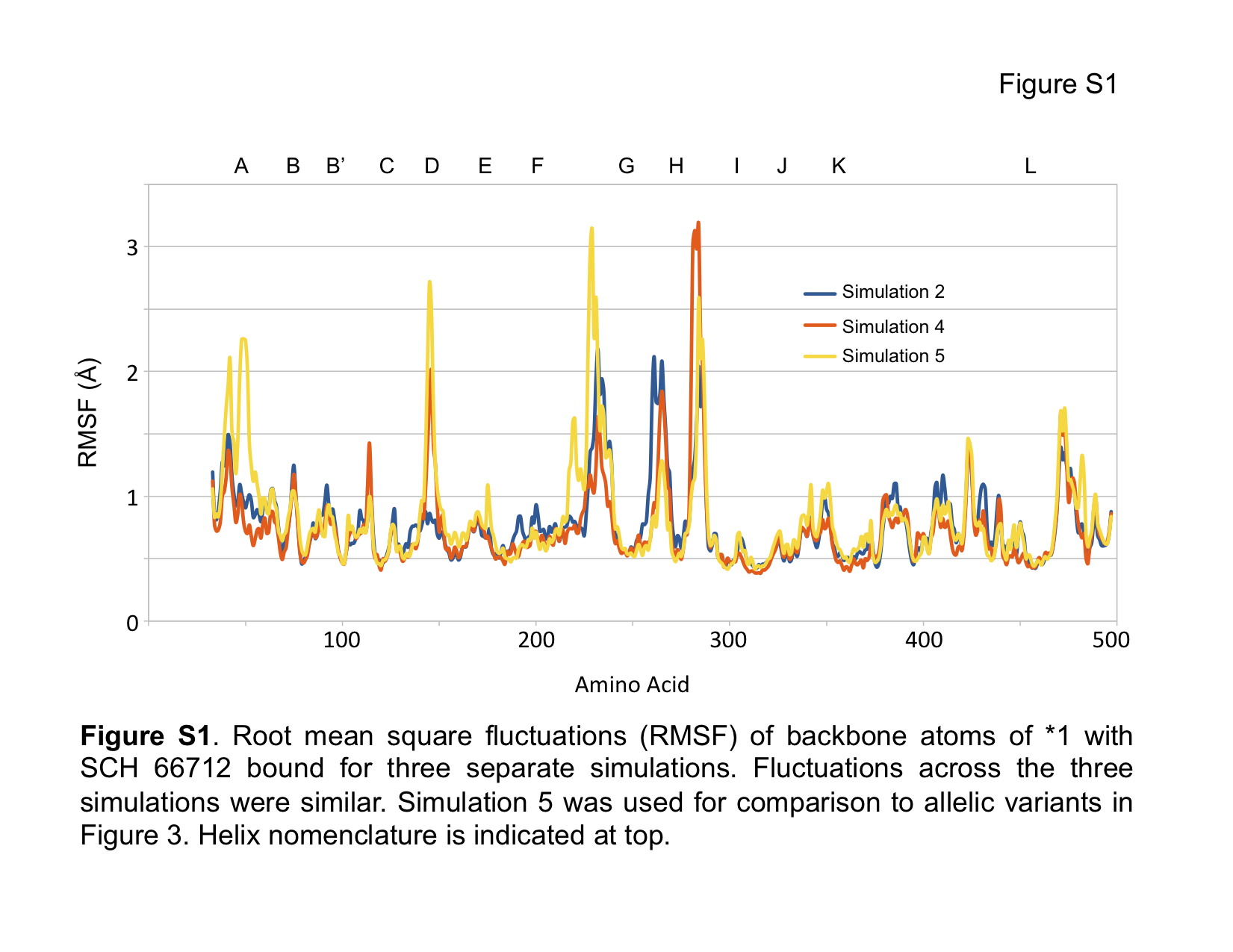

Supplement: Figure S1 — Root mean square fluctuations (RMSF) of backbone atoms of *1 with SCH 66712 bound for three separate simulations. Fluctuations across the three simulations were similar. Simulation 5 was used for comparison to allelic variants in Figure 3. Helix nomenclature is indicated at top. (TIFF) [file pone.0108607.s001.tiff]

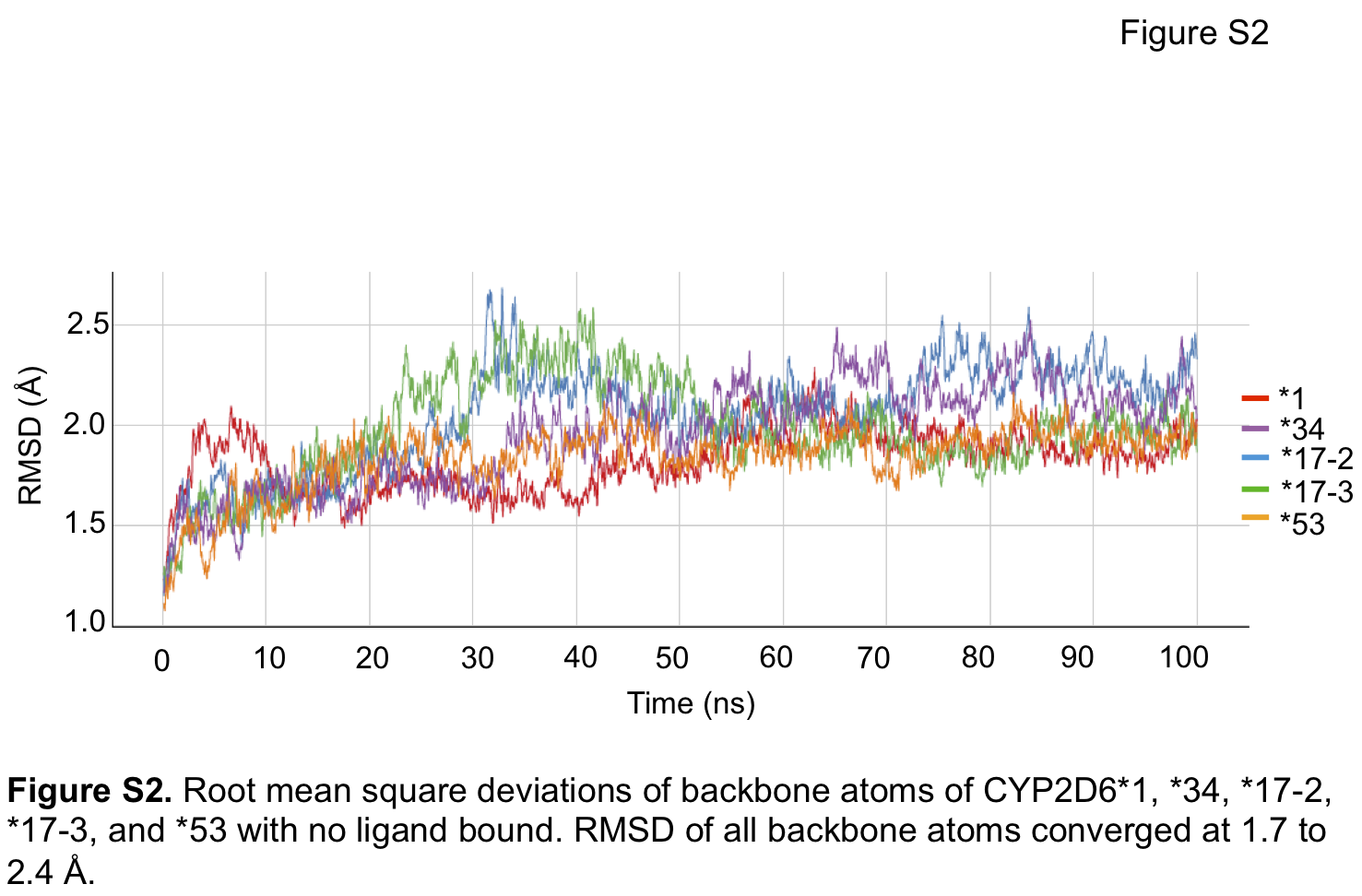

Supplement: Figure S2 — Root mean square deviations of backbone atoms of CYP2D6*1, *34, *17-2, *17-3, and *53 with no ligand bound. RMSD of all backbone atoms converged at 1.7 to 2.3 Å. (TIFF) [file pone.0108607.s002.tiff]

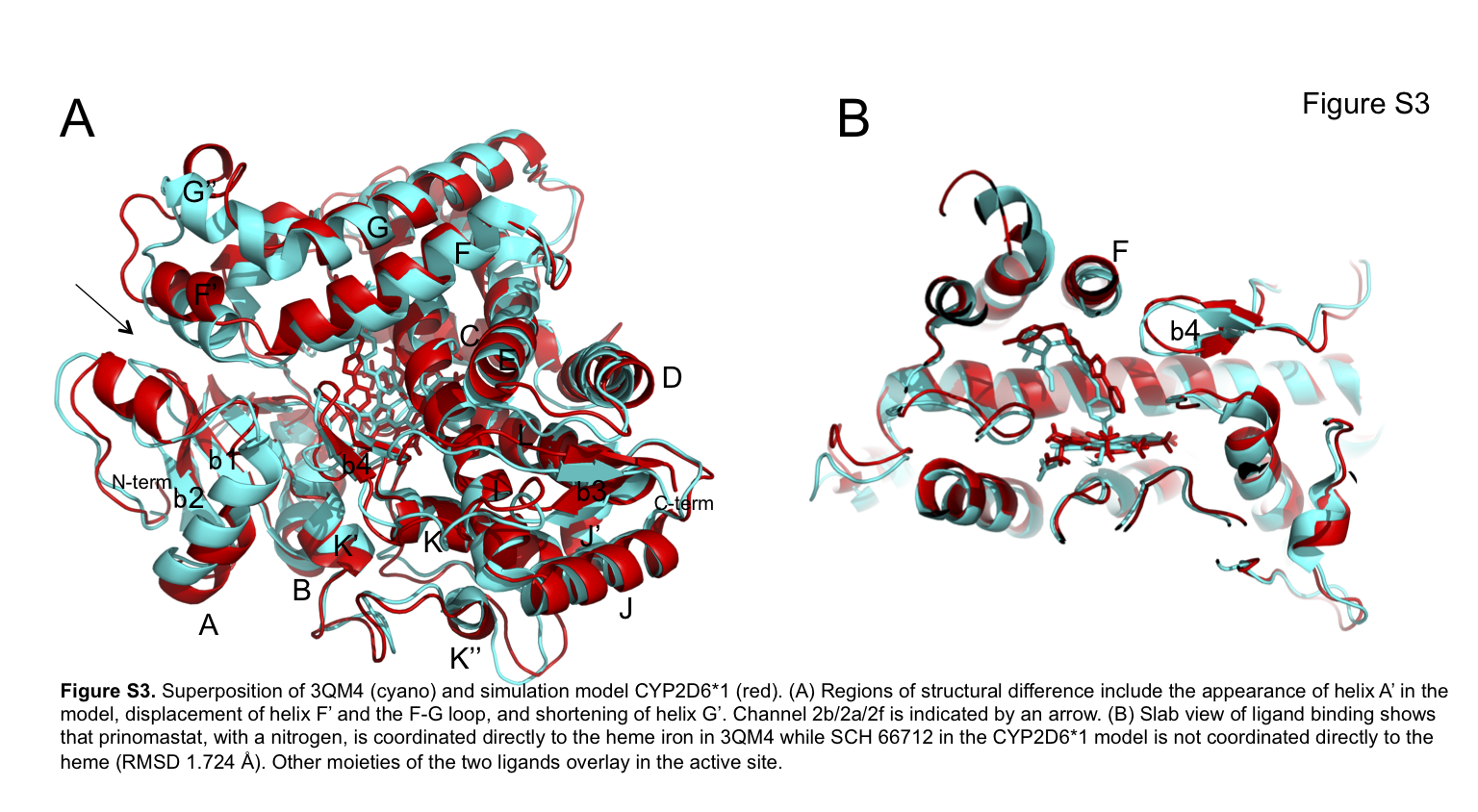

Supplement: Figure S3 — Superposition of 3QM4 (cyano) and simulation model CYP2D6*1 (red). (A) Regions of structural difference include the appearance of helix A′ in the model, displacement of helix F′ and the F-G loop, and shortening of helix G′. Channel 2b/2a/2f is indicated by an arrow. (B) Slab view of ligand binding shows that prinomastat, with a nitrogen, is coordinated directly to the heme iron in 3QM4 while SCH 66712 in the CYP2D6*1 model is not coordinated directly to the heme (RMSD 1.724 Å). Other moieties of the two ligands overlay in the active site. (TIFF) [file pone.0108607.s003.tiff]

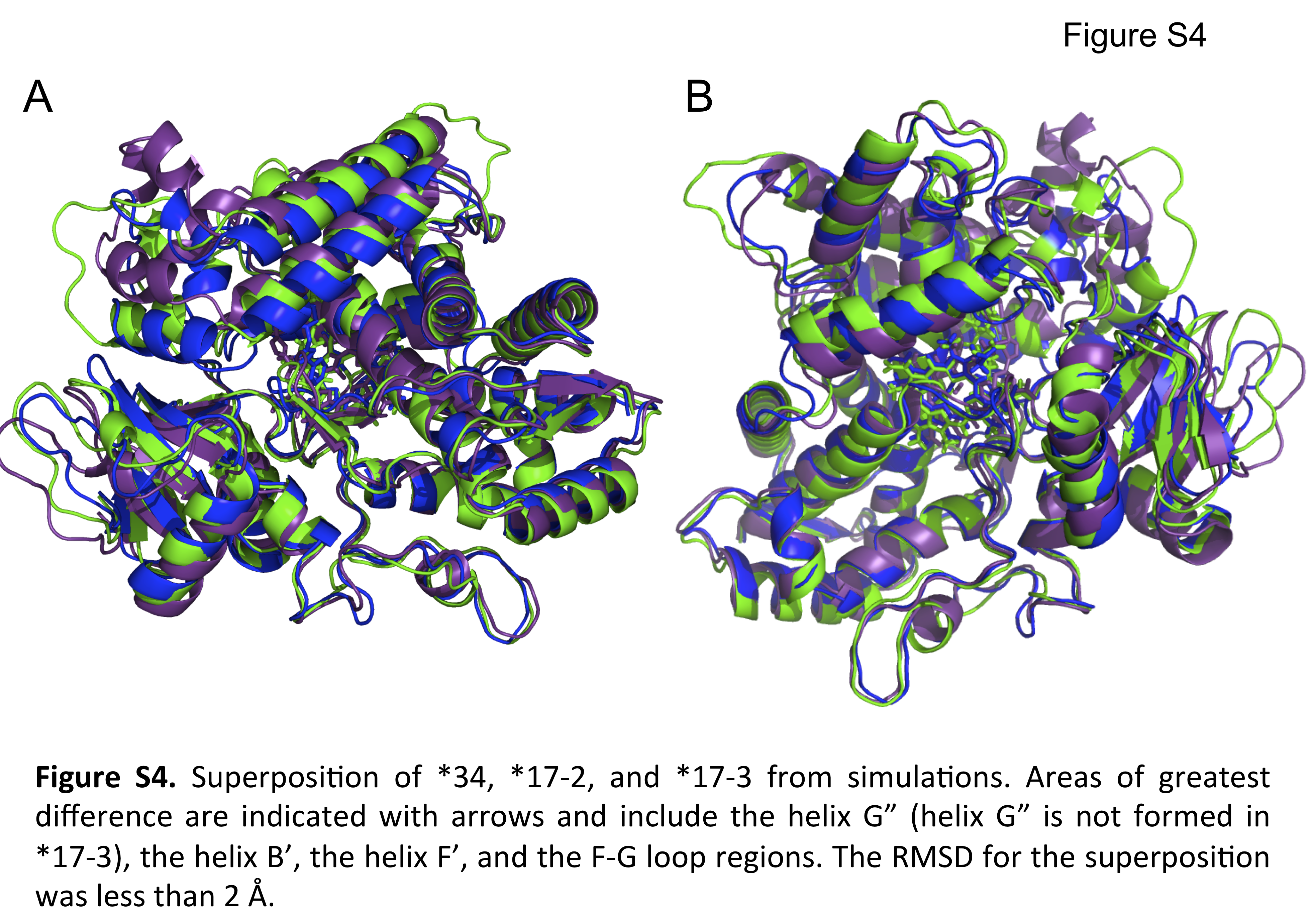

Supplement: Figure S4 — Superposition of *34, *17-2, and *17-3 from simulations. Areas of greatest difference are indicated with arrows and include the helix G″ (helix G″ is not formed in *17-3), the helix B′, the helix F′, and the F-G loop regions. The RMSD for the superposition was less than 2 Å. (TIFF) [file pone.0108607.s004.tiff]

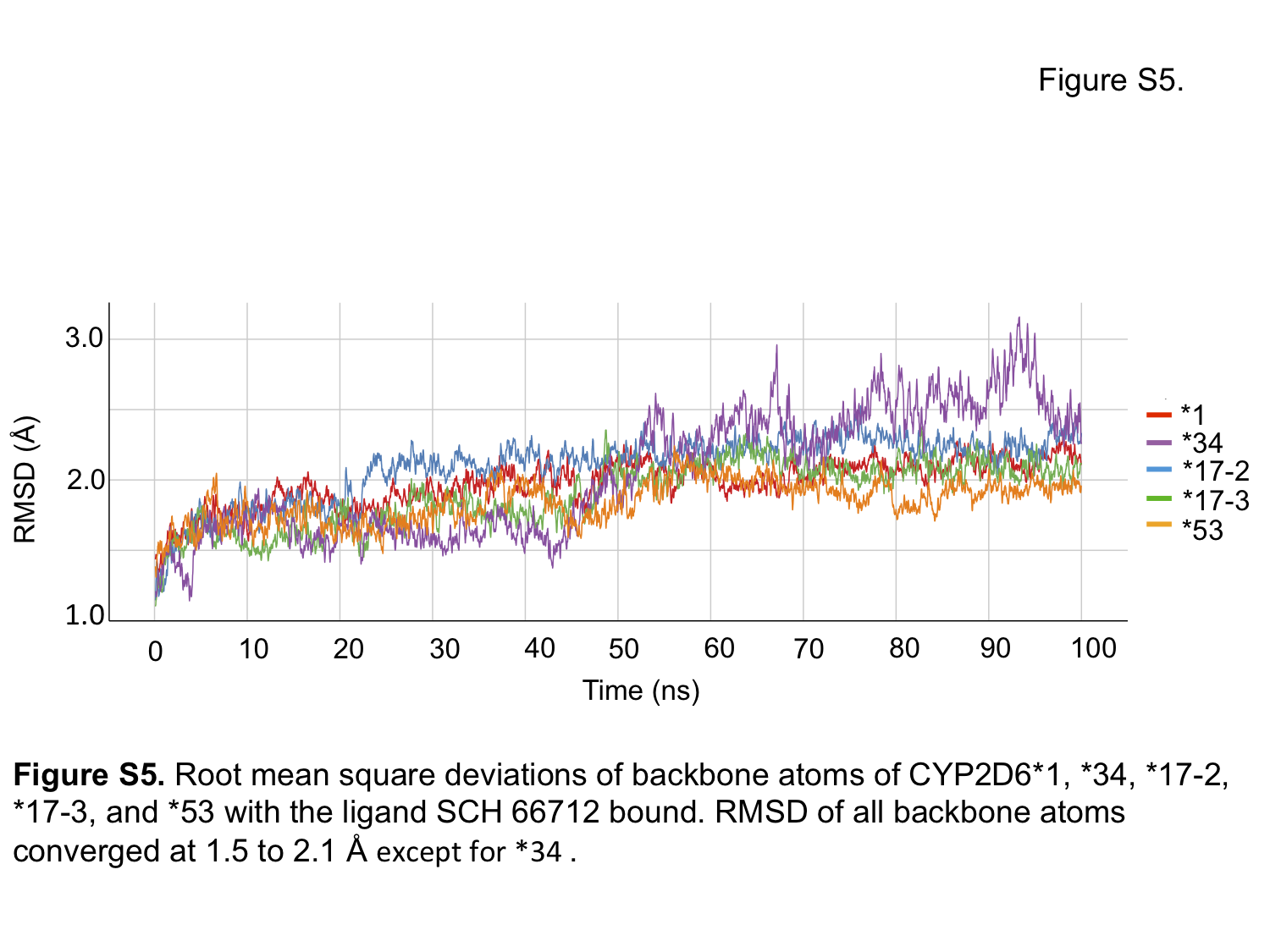

Supplement: Figure S5 — Root mean square deviations of backbone atoms of CYP2D6*1, *17-2, *17-3, *34, and *53 with the ligand SCH 66712 bound. RMSD of all backbone atoms converged at 1.5 to 2.1 Å except for *34. (TIFF) [file pone.0108607.s005.tiff]

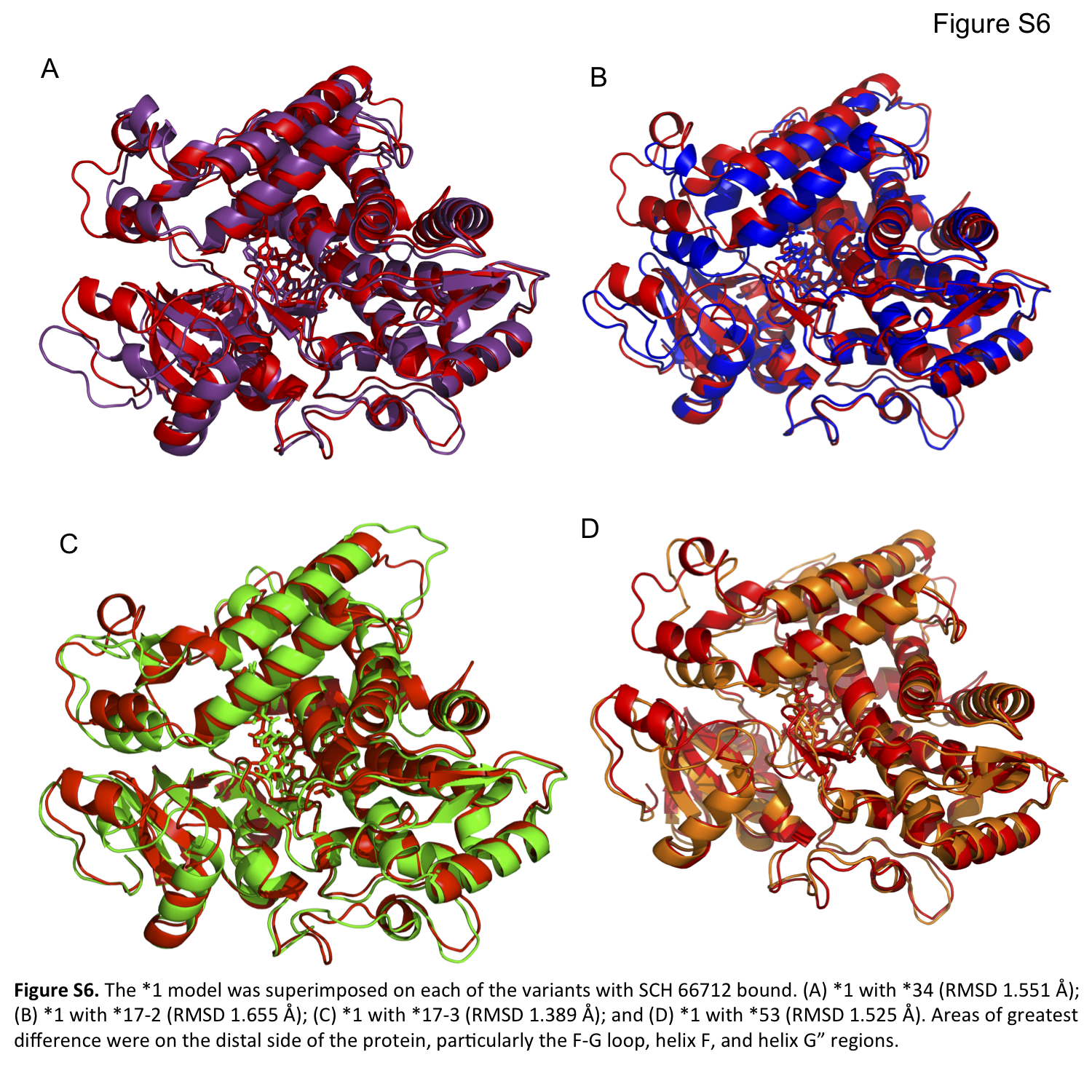

Supplement: Figure S6 — The *1 model was superimposed on each of the variants with SCH 66712 bound. (A) *1 with *34 (RMSD 1.551 Å); (B) *1 with *17-2 (RMSD 1.655 Å); (C) *1 with *17-3 (RMSD 1.389 Å); and (D) *1 with *53 (RMSD 1.525 Å). Areas of greatest difference were on the distal side of the protein, particularly the F-G loop, helix F, and helix G″ regions. (TIFF) [file pone.0108607.s006.tiff]

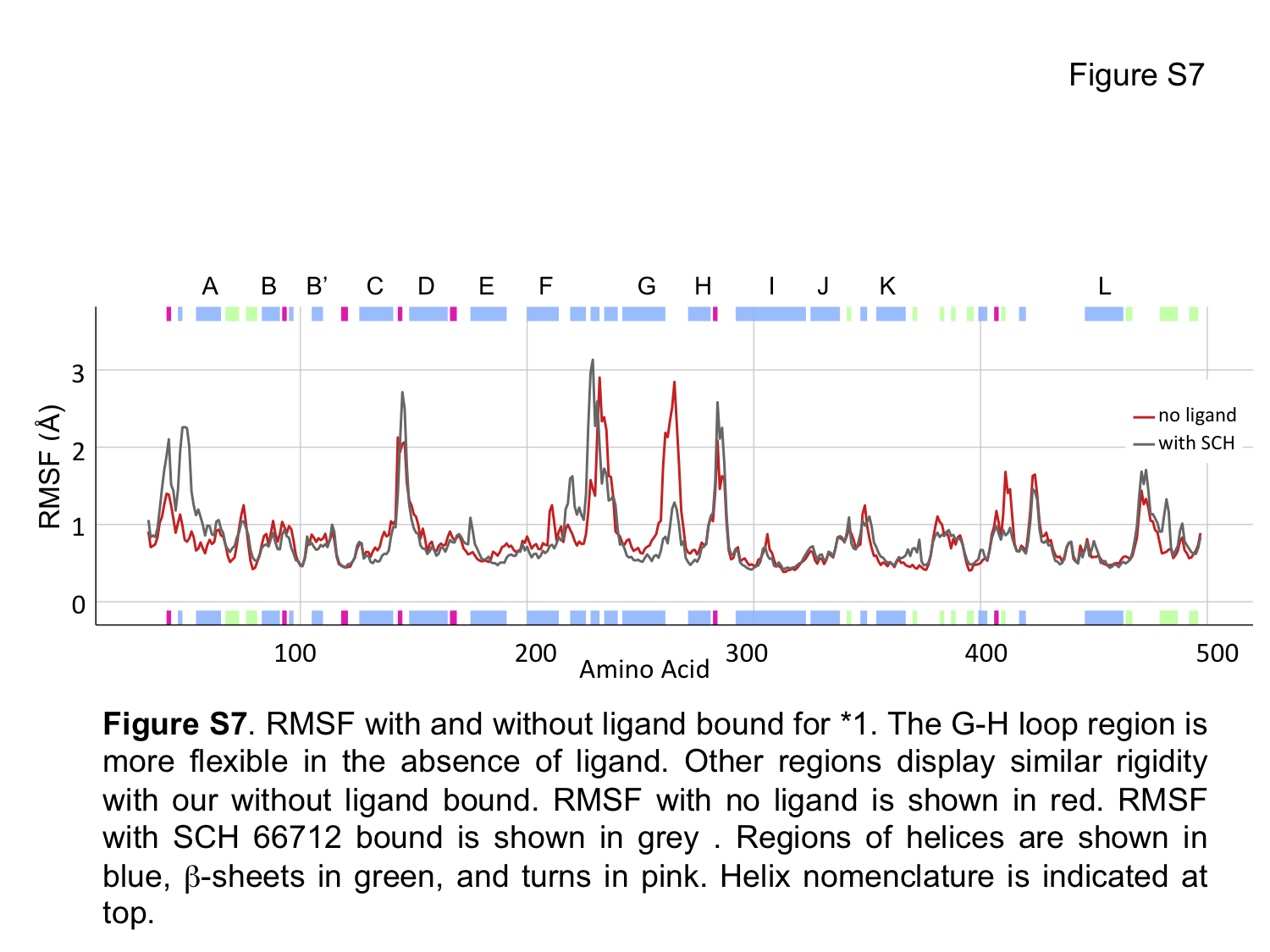

Supplement: Figure S7 — RMSF with and without ligand bound for *1. The G-H loop region is more flexible in the absence of ligand. Other regions display similar rigidity with our without ligand bound. RMSF with no ligand is shown in red. RMSF with SCH 66712 bound is shown in grey. Regions of helices are shown in blue, β-sheets in green, and turns in pink. Helix nomenclature is indicated at top. (TIFF) [file pone.0108607.s007.tiff]

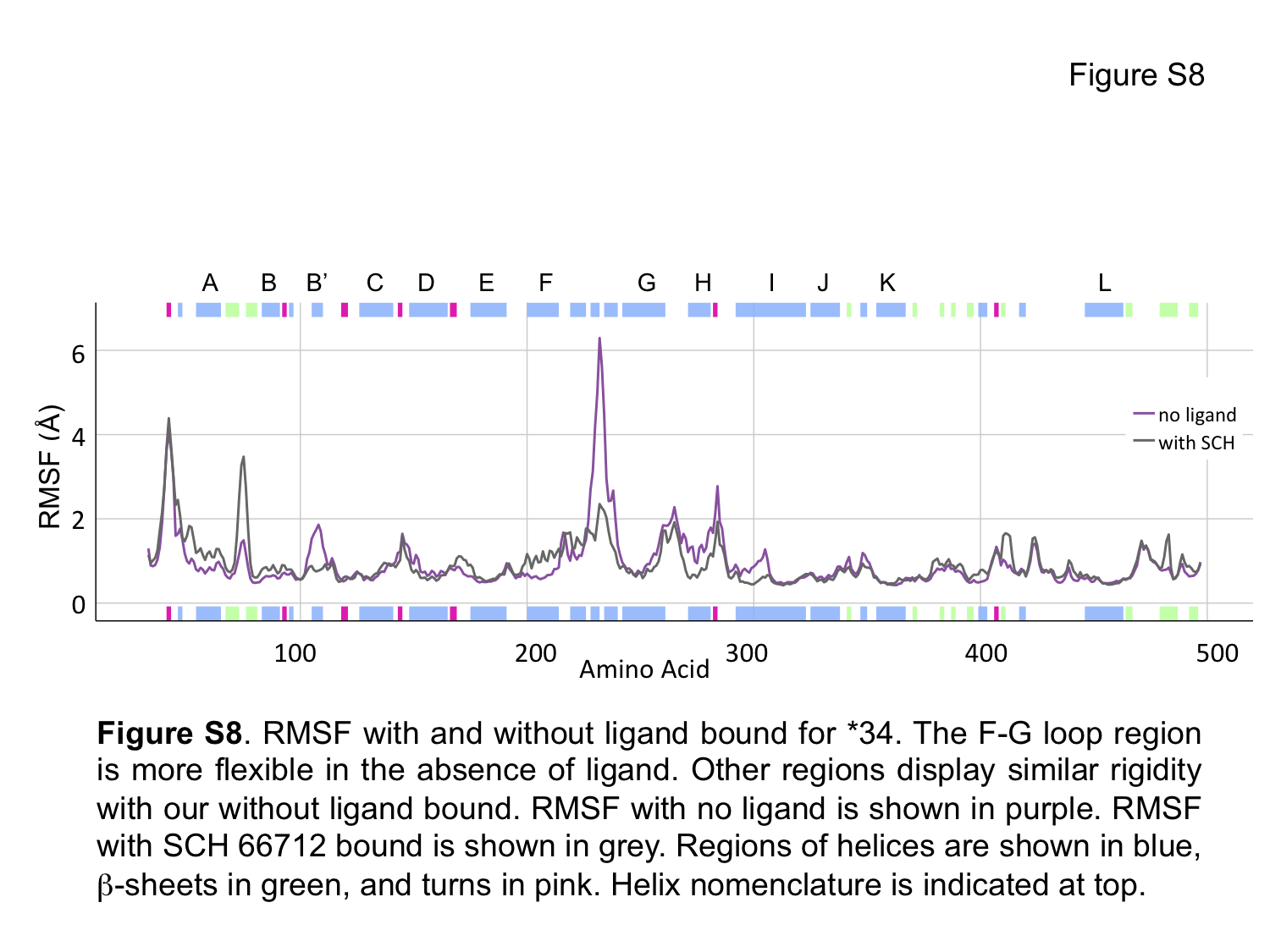

Supplement: Figure S8 — RMSF with and without ligand bound for *34. The F-G loop region is more flexible in the absence of ligand. Other regions display similar rigidity with our without ligand bound. RMSF with no ligand is shown in purple. RMSF with SCH 66712 bound is shown in grey. Regions of helices are shown in blue, β-sheets in green, and turns in pink. Helix nomenclature is indicated at top. (TIFF) [file pone.0108607.s008.tiff]

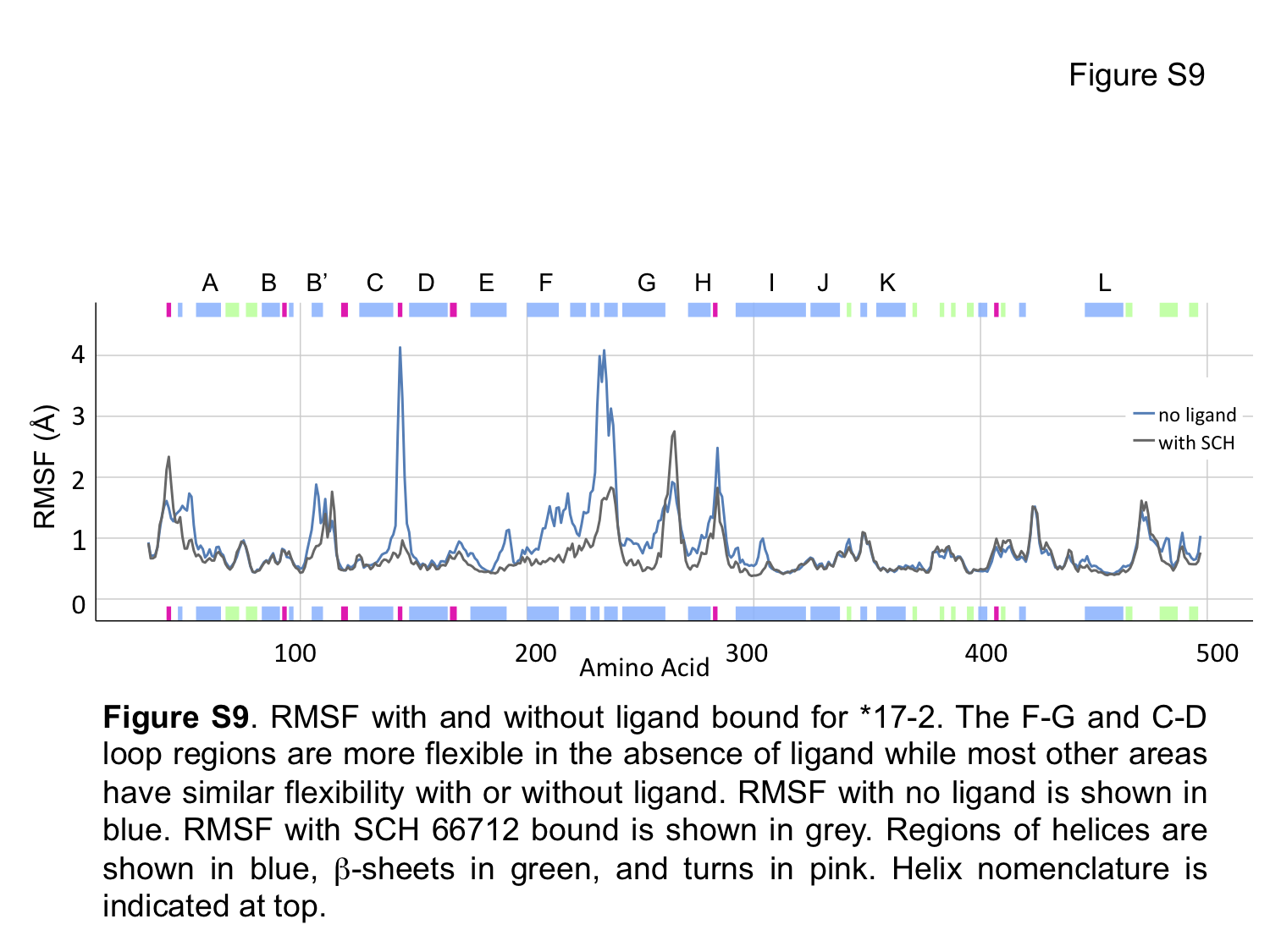

Supplement: Figure S9 — RMSF with and without ligand bound for *17-2. The F-G and C-D loop regions are more flexible in the absence of ligand while most other areas have similar flexibility with or without ligand. RMSF with no ligand is shown in blue. RMSF with SCH 66712 bound is shown in grey. Regions of helices are shown in blue, β-sheets in green, and turns in pink. Helix nomenclature is indicated at top. (TIFF) [file pone.0108607.s009.tiff]

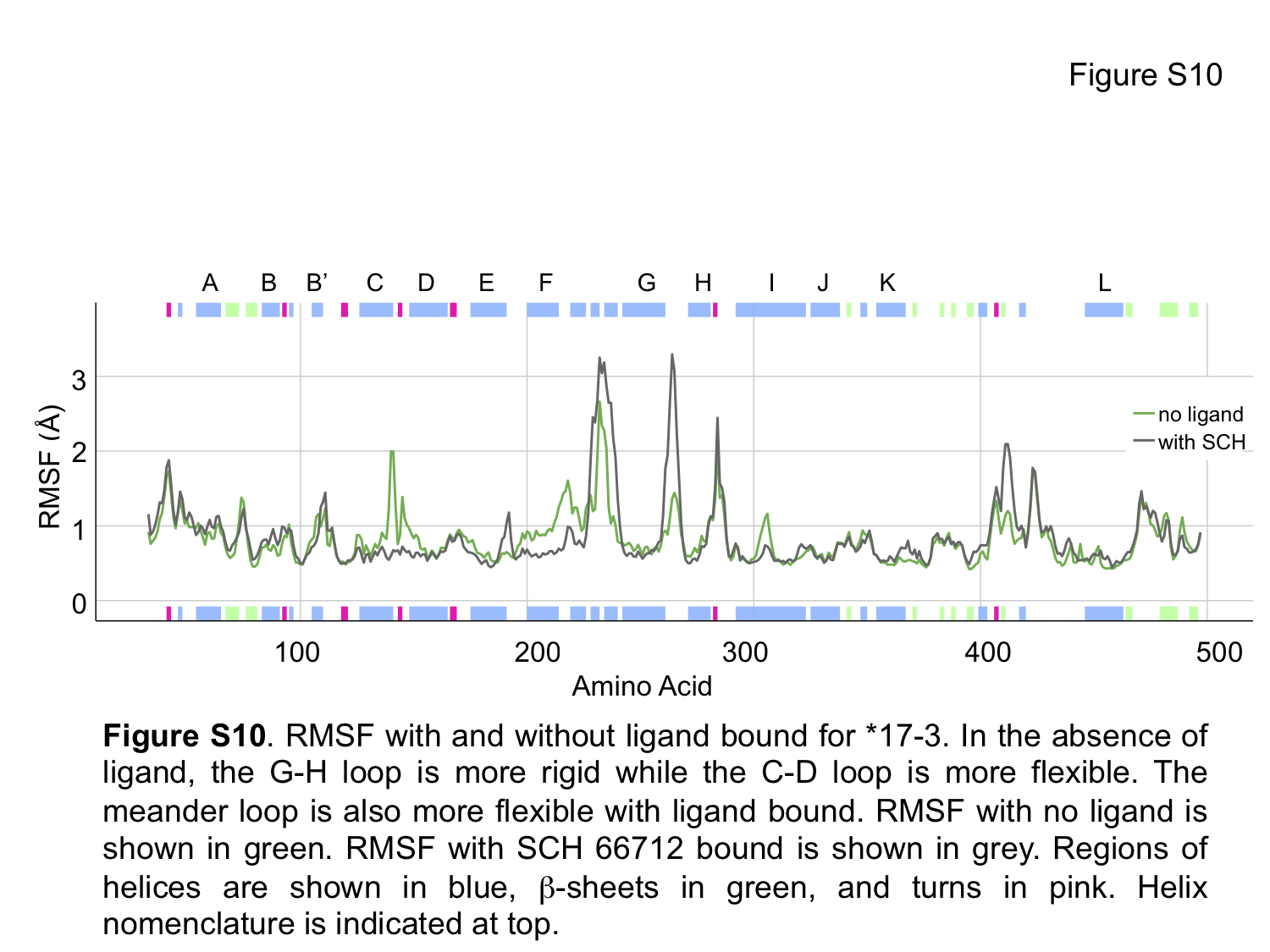

Supplement: Figure S10 — RMSF with and without ligand bound for *17-3. In the absence of ligand, the G-H loop is more rigid while the C-D loop is more flexible. The meander loop is also more flexible with ligand bound. RMSF with no ligand is shown in green. RMSF with SCH 66712 bound is shown in grey. Regions of helices are shown in blue, β-sheets in green, and turns in pink. Helix nomenclature is indicated at top. (TIFF) [file pone.0108607.s010.tiff]

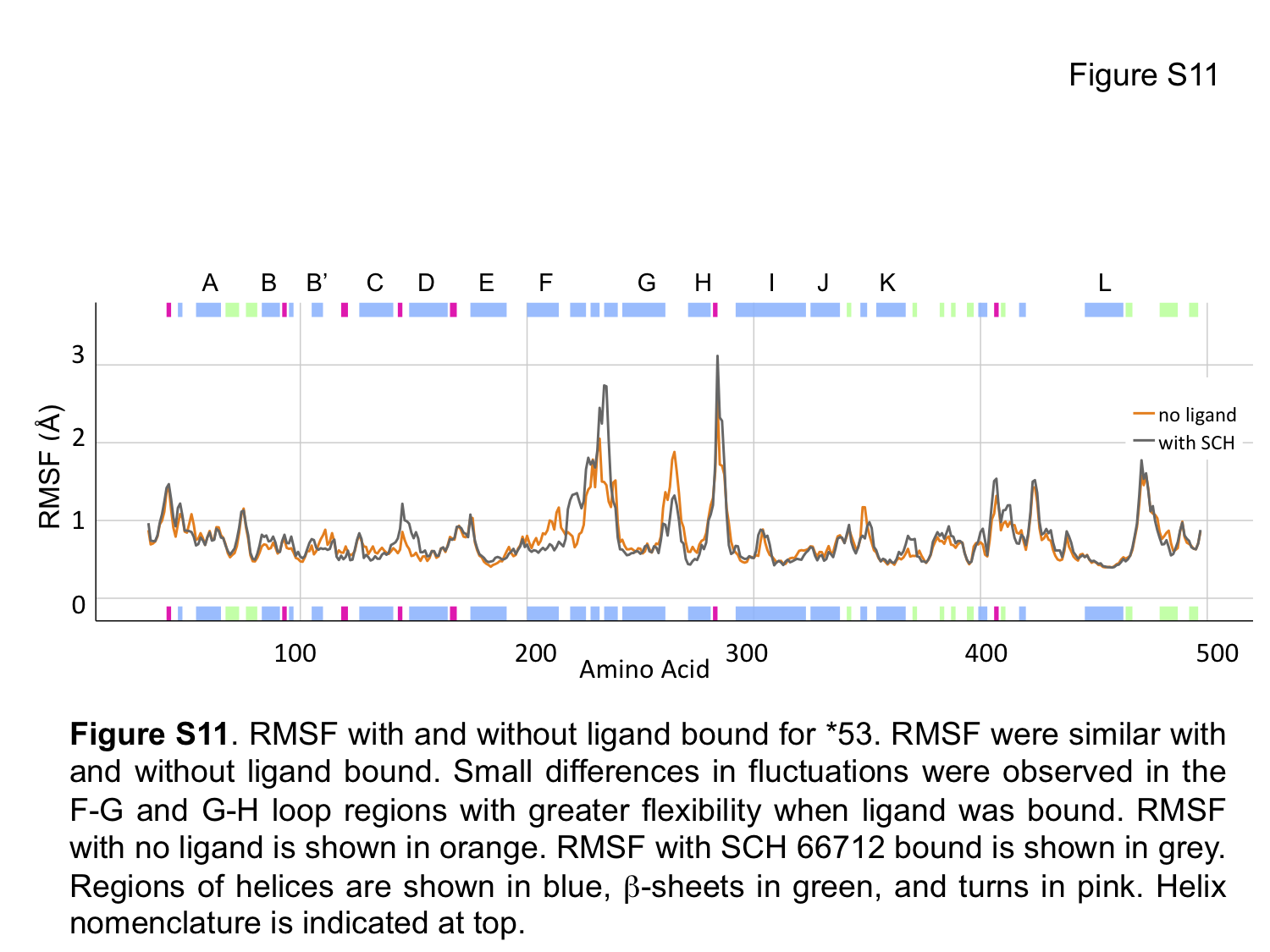

Supplement: Figure S11 — RMSF with and without ligand bound for *53. RMSF were similar with and without ligand bound. Small differences in fluctuations were observed in the F-G and G-H loop regions with greater flexibility when ligand was bound. RMSF with no ligand is shown in orange. RMSF with SCH 66712 bound is shown in grey. Regions of helices are shown in blue, β-sheets in green, and turns in pink. Helix nomenclature is indicated at top. (TIFF) [file pone.0108607.s011.tiff]

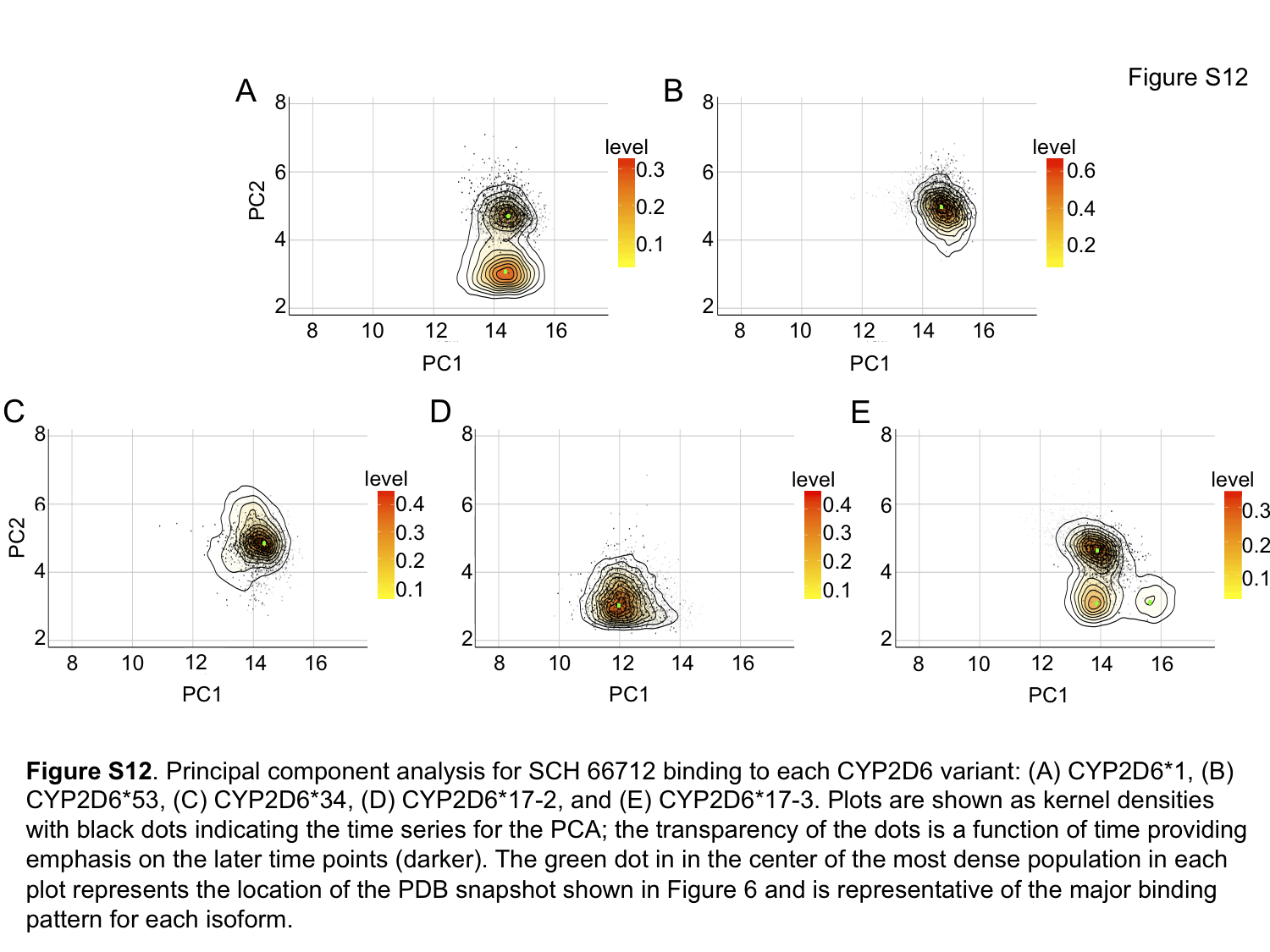

Supplement: Figure S12 — Principal component analysis for SCH 66712 binding to each CYP2D6 variant: (A) CYP2D6*1, (B) CYP2D6*53, (C) CYP2D6*34, (D) CYP2D6*17-2, (E) CYP2D6*17-3. Plots are shown as kernel densities with black dots indicating the time series for the PCA; the transparency of the dots is a function of time providing emphasis on the later time points (darker). The green dot in each plot represents the location of the PDB snapshot shown in Figure 4B as representative of the binding patterns for each isoform. (TIFF) [file pone.0108607.s012.tiff]

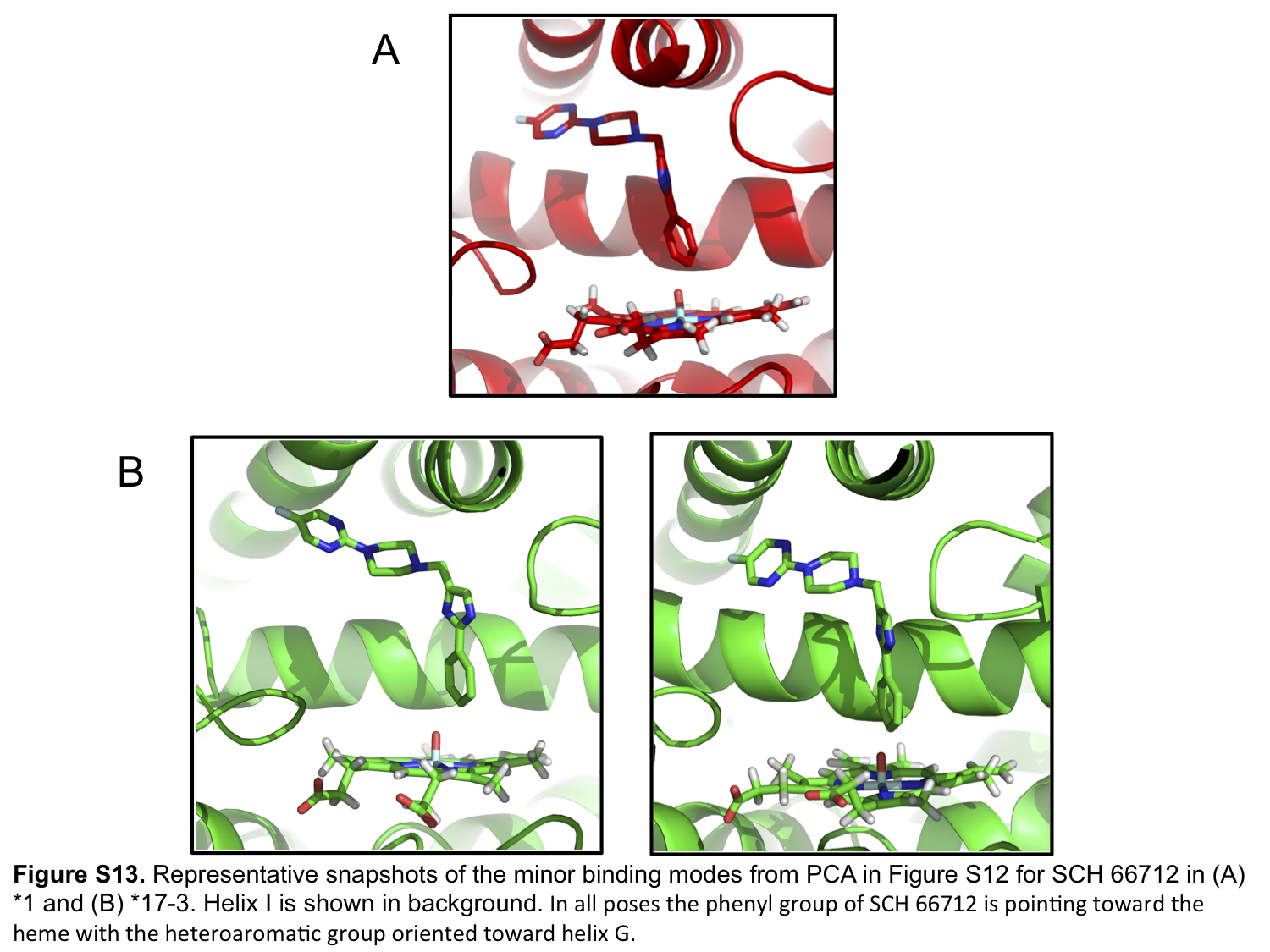

Supplement: Figure S13 — Representative snapshots of the minor binding modes from PCA in Figure S12 for SCH 66712 in (A) *1 and (B) *17-3. Helix I is shown in background. In all poses the phenyl group of SCH 66712 is pointing toward the heme with the heteroaromatic group oriented toward helix G. (TIFF) [file pone.0108607.s013.tiff]
