# Supplementary material for: Molecular Dynamics of CYP2D6 Polymorphisms in the Absence and Presence of a Mechanism-Based Inactivator Reveals Changes in Local Flexibility and Dominant Substrate Access Channels
Source: PLoS One. 2014 Oct 6;9(10):e108607. doi: 10.1371/journal.pone.0108607 (PMC4186923; doi:10.1371/journal.pone.0108607)
Supplement: Scheme S1 — C programming language script used for identification of the coordinates of the oxygen and iron of Compound I and for defining the starting point for CAVER analysis. (DOCX) [file pone.0108607.s014.docx]

**Scheme S1.** C programming language script used for identification of the coordinates of the oxygen and iron of Compound I and for defining the starting point for CAVER analysis.

C script for locating the start position for CAVER analysis:

#import "vector.h"

#import <stdio.h>

#import <stdlib.h>

#import <string.h>

#define SAME 0

/*

* Utility to calculate a starting point from a pdb file.

* The starting point is located 4 angstroms above the oxygen at the center of the heme.

* Requires that the oxygen is named "O1 " and the iron is named "FE ".

* These names are located in columns 13-16, as defined in the PDB file format.

* Uses vector arithmatic to accomplish the task.

* Prints the calculated starting point to standard output.

*

* Usage: ./startLoc path/to/pdb/foo.pdb

*/

int main(int argc, char* argv[])

{

FILE* fptr = fopen(argv[1], "r");

int len;

char line[100];

Vector *o, *fe, *neg, *dir, *offset, *startingPt;

o = (Vector*) malloc(sizeof(Vector));

fe = (Vector*) malloc(sizeof(Vector));

while (fgets(line, 100, fptr) != NULL)

{

if(strncmp(line+13, "FE ", 3)==SAME)

{

sscanf(line+32, "%lf %lf %lf", &fe->x, &fe->y, &fe->z);

}else if(strncmp(line+13, "O1 ", 3)==SAME)

{

sscanf(line+32, "%lf %lf %lf", &o->x, &o->y, &o->z);

}

}

/* dir = o - fe */

neg = multiply(fe, -1);

dir = add(o, neg);

/* make of length 4 */

dir = normalize(dir);

offset = multiply(dir, 4);

/* add to the oxygen */

startingPt = add(o, offset);

printf("%f %f %f\n", startingPt->x, startingPt->y, startingPt->z);

fclose(fptr);

return 0;

}

VECTOR H script:

#ifndef VECTOR_H

#define VECTOR_H

typedef struct vector_t

{

double x;

double y;

double z;

/* data */

} Vector;

double magnitude(Vector* v);

Vector* normalize(Vector* v);

Vector* multiply(Vector* v, double mag);

Vector* add(Vector* v1, Vector* v2);

#endif

VECTOR C script:

#import <math.h>

#import <stdlib.h>

#import "vector.h"

double magnitude(Vector* v)

{

return sqrt(v->x*v->x + v->y*v->y + v->z*v->z);

}

Vector* normalize(Vector* v)

{

double mag = magnitude(v);

Vector* ret = multiply(v, 1/mag);

return ret;

}

Vector* multiply(Vector* v, double mag)

{

Vector* ret = (Vector*) malloc(sizeof(Vector));

ret->x = v->x*mag;

ret->y = v->y*mag;

ret->z = v->z*mag;

return ret;

}

Vector* add(Vector* v1, Vector* v2)

{

Vector* ret = (Vector*) malloc(sizeof(Vector));

ret->x = v1->x+v2->x;

ret->y = v1->y+v2->y;

ret->z = v1->z+v2->z;

return ret;

}
